# Protocol for a scoping review of PTSD and minority stress interventions for LGBTQIA + adults

**DOI:** 10.1007/s44202-025-00355-2

**Published:** 2025-07-17

**Authors:** Eve A. Rosenfeld, Nadia Malek, McKenzie Lockett, Donovan Edward, Vaughan Hooper, Kelly L. Harper, Cara Herbitter, Sheila M. Thompson, Alexis Ceja, Muska Saty, Cindy J. Chang, Elizabeth N. Collazo, Laura Ong, Christopher Stave, Alex Cudd, Alec O’Reilly, Nicholas A. Livingston

**Affiliations:** 1 Dissemination and Training Division, National Center for PTSD, VA Palo Alto Health Care System, Palo Alto, CA USA; 2 Department of Psychiatry and Behavioral Sciences, Stanford University, Stanford,, CA USA; 3 Psychology Department, Arizona State University, Tempe, AZ USA; 4 Department of Psychology, Georgia Southern University, Statesboro, GA USA; 5 Palo Alto Veterans Institute for Research, Palo Alto, CA USA; 6 Department of Psychology, Oregon State University, Corvallis, OR USA; 7 National Center for PTSD, Behavioral Science Division, VA Boston Healthcare System, Boston, MA USA; 8 Department of Psychiatry, Boston University Chobanian & Avedisian School of Medicine, Boston, MA USA; 9 VA Boston Healthcare System, Boston, MA USA; 10 Department of Mathematics, California State University, Fullerton, CA USA; 11 VA San Diego Healthcare System, San Diego, CA USA; 12 Department of Psychiatry, University of California, San Diego, CA USA; 13 Department of Psychology, Northern Illinois University, DeKalb, IL USA; 14 Lane Medical Library, Stanford University, Stanford, CA USA; 15 Midwestern University Medical School, Downers Grove, IL USA

**Keywords:** Scoping review, LGBTQIA +, Minority stress, Posttraumatic stress disorder, Sexual and gender minority

## Abstract

**Background:**

LGBTQIA + people experience trauma and posttraumatic stress disorder (PTSD) at higher rates than cisgender heterosexual people, in addition to experiencing minority stress. There remains a dearth of research on appropriate PTSD interventions and minority stress interventions for LGBTQIA + people. However, the scope of the literature on neither PTSD interventions nor minority stress interventions for LGBTQIA + adults has ever been reviewed. Furthermore, research on PTSD-focused and minority stress-focused interventions remains relatively siloed, despite the link between minority stress and PTSD symptoms. The proposed scoping review aims to: (1) describe the scope of the current literature, chart available data, and synthesize findings, (2) collate information on existing PTSD and minority stress interventions for LGBTQIA + adults, and (3) identify gaps in the literature and directions for future research.

**Methods:**

Research produced since 2000 on psychological, psychotherapeutic, and behavioral interventions for PTSD, minority stress, or both within the LGBTQIA + adult population will be reviewed. This protocol follows the Preferred Reporting Items for Systematic reviews and Meta-Analyses for Scoping Reviews (PRISMA-ScR).

**Results:**

The search yielded a total of 6818 results. Following deduplication, a total of 4945 results remained. Title/abstract screening will be followed by full-text review and data coding, charting, and mapping.

**Conclusions:**

This scoping review will be the first to describe the state of the literature and synthesize information on both PTSD and minority stress interventions for LGBTQIA + adults. Findings may also highlight promising interventions and treatment components. Information gleaned may inform future adaptations of existing interventions and development of new interventions for LGBTQIA + adults experiencing PTSD and/or minority stress.

**Public significance statement:**

This paper discusses the planned steps for an ongoing scoping review. The scoping review will provide an overview of treatments for PTSD and for minority stress for LGBTQIA + adults.

**Supplementary Information:**

The online version contains supplementary material available at 10.1007/s44202-025-00355-2.

Posttraumatic stress disorder (PTSD) is particularly prevalent among lesbian, gay, bisexual, transgender, queer, intersex, asexual, and other minoritized sexual orientation and gender identity (LGBTQIA +) individuals [33]. While estimates indicate 7–8% PTSD prevalence among the general population, up to 48% of LGBTQIA + individuals meet diagnostic criteria for PTSD [30, 31]. The elevated prevalence of PTSD among LGBTQIA + individuals is driven in part by disproportionate exposure to traumatic events [3, 6, 15, 49], particularly for interpersonal trauma (i.e., traumatic events involving at least one perpetrator and victim [3, 49, 53]. Interpersonal trauma is associated with greater risk of developing PTSD compared to other types of trauma [22, 24]. Further, LGBTQ + individuals face unique risk factors based on their marginalization that contribute to PTSD prevalence and severity.

Minority stress, or the psychosocial stress experienced by members of a marginalized group, is a key contributing factor in the increased prevalence and severity of mental health conditions among LGBTQIA + individuals [29, 36, 38]. Minority stress was initially defined as a construct by Brooks [10], whose work outlining interpersonal, social, and economic stressors that disparately impact sexual minority women set the stage for later conceptualizations of minority stress [44]. The minority stress model [38] distinguishes between distal stressors, which refer to experiences of prejudice and discrimination (e.g., harassment, microaggressions) perpetrated by other people and institutions, and proximal stressors, which describe the internalization of identity-based stressors including concealing one’s identity and experiences of internalized stigma. According to the minority stress model, distal stressors give rise to internal stress processes (i.e., proximal stressors). For example, due to previous experiences of prejudice, LGBTQIA + people often have an acute awareness of how others treat them due to their minority status, marked by an underlying expectation of rejection [21, 45]. To avoid rejection, some LGBTQIA + individuals navigate the world by hiding or concealing their sexual and/or gender identities [11, 54]. Stress caused by the concealment of identities has been noted as a proximal stressor due in part to the hypervigilance of maintaining the façade created. Additionally, in response to distal stressors, LGBTQIA + individuals may internalize negative beliefs and stereotypes about their identity (i.e., internalized stigma; [39]).

Understanding and addressing the complex interrelations of minority stress, trauma, and PTSD is critically important to adequately meet the mental health needs of LGBTQIA + trauma survivors. Minority stress and PTSD symptoms are interconnected in several ways. First, minority stress can increase vulnerability to the effects of trauma exposure. Cardona et al. [14] theorized that minority stressors can act as chronic traumatic invalidation and thereby cause emotion dysregulation. Hatzenbuehler [28] similarly proposed that stigma-related stress can generalize to broader deficits in emotion regulation, social/interpersonal functioning, and cognitive processes, in turn heightening vulnerability to psychopathology. Second, emerging research indicates that LGBTQIA + individuals view their experiences of trauma and minority stress as inextricably intertwined [7, 56]. Third, certain forms of distal minority stress (e.g., identity-based physical and sexual violence) qualify as Criterion A traumas as defined by the DSM-5 [2], increasing the proportion of LGBTQIA + people eligible for a PTSD diagnosis [25]. Fourth, minority stressors that do not meet Criterion A trauma (e.g., familial rejection, verbal abuse) cause distress and can exacerbate PTSD symptoms that are otherwise unrelated to one’s gender or sexual minority status [31]. For example, internalized heterosexism (a specific form of internalized stigma) exacerbates PTSD symptom severity over time [23]. Fifth, responses to minority stress may present similarly to PTSD symptoms (e.g., hypervigilance, intrusive thoughts), further complicating the recovery process. Whereas PTSD symptoms are, by definition, connected to a past event [2], minority stress symptoms can stem from both past events *and* chronic everyday stressors (e.g., discriminatory laws, persistent misgendering) that entail ongoing threat; psychotherapy that addresses trauma without addressing ongoing minority stress may thus be insufficient in resolving PTSD symptoms [31]. LGBTQIA + affirmative psychotherapy also requires considering that some minority stress responses may be adaptive to protect against ongoing risks (e.g., adaptive vigilance; [31]). Though the empirical base for LGBTQIA + affirmative care practices and minority stress interventions is developing [40, 41], very few of these interventions are specifically focused on trauma-exposed or traumatized LGBTQIA + people. Therefore, the interaction of minority stress, trauma, and PTSD symptoms has important implications for the treatment of PTSD (and vice versa), yet the literature evaluating PTSD treatments and the literature evaluating minority stress interventions for LGBTQ + adults remain siloed.

In addition, there are other factors that can influence the interplay and impact of minority stress, trauma, and PTSD for LGBTQIA + people. For example, racially and ethnically minoritized people are more likely to be exposed to trauma [35], yet less likely to have access to mental health services [18]. Racially and ethnically minoritized LGBTQIA + individuals often face minority stressors related to both their racial and ethnic identities as well as their gender and sexual identities, begetting a higher burden of stress. Similar to sexual and gender minority stressors, race-based stressors are often experienced as traumatic [58] and can contribute to the development and maintenance of PTSD [8, 34]. Further, racially and ethnically minoritized LGBTQIA + people often experience racism and exclusion from white LGBTQIA + people, but may also experience heterosexism from their racial and ethnic in-group members, creating a barrier to social support [20]. In particular, Black transgender women often face high levels of trauma exposure, discrimination, and barriers to healthcare [47, 48]. Non-westernized conceptualizations of queerness, like Two-Spirit identities in indigenous North American communities or Fa’afafine identity in Somoa, also experience unique marginalization, yet these groups have received little scientific attention until recently [5, 9, 13].

Potentially due to the heightened psychological distress associated with marginalization, LGBTQIA + individuals use more mental health care than their cisgender, heterosexual peers [17, 26, 43, 49]. Despite this, we know little about their use of PTSD-specific treatments. Most PTSD treatment effectiveness and efficacy studies have not reported on sexual orientation and gender identity [32] and studies on mental health care utilization comparing LGBTQIA + and non-LGBTQIA + communities have not reported on PTSD-specific care (e.g., [17, 26, 43, 49]). Additionally, despite high mental health service utilization, LGBTQIA + individuals experience stigma and discrimination in health care, have negative experiences with mental health treatment, and report significant unmet mental health treatment needs [1, 12, 37, 46, 50, 51]. For example, LGBTQIA + individuals are approximately two times more likely to report an unmet mental health need compared to their cisgender, heterosexual counterparts [16, 52]. LGBTQIA + individuals also report frequently avoiding mental health services due to concerns of provider-perpetrated stigmatization of their LGBTQIA + identity [51] and have more negative perceptions of mental health care (e.g., lower satisfaction) compared to cisgender, heterosexual peers [4]. Disparities in mental health care quality and access likely stem in part from insufficient training and education in LGBTQIA + specific concerns among mental health providers [19].

Current mental health services thus appear inadequately tailored to address the unique needs and lived experiences of LGBTQIA + patients. Indeed, a recent study demonstrated that higher levels of discrimination experiences in day-to-day life are associated with lower satisfaction with and helpfulness of mental healthcare among bisexual individuals [27]. Specific to PTSD treatment, research suggests that LGBTQIA + people who received counseling for PTSD experienced more microaggressions in treatment and a greater number of barriers to treatment compared to non-PTSD focused counseling [1]. Additionally, greater experiences of microaggressions in treatment explained lower satisfaction with treatment [1]. Taken together, these findings highlight the critical need for culturally-tailored interventions for LGBTQIA + adults with symptoms of PTSD and minority stress and the integration of evidence-based components from each.

Given the high prevalence of PTSD and experiences of minority stress among the LGBTQIA + community, there is an urgent need for identity-affirming interventions that address trauma-related symptomatology. However, research in the following areas is often siloed: interventions for PTSD, interventions for minority stress, and sexual and gender minority identity-affirming care. As such, it is difficult to determine whether existing interventions sufficiently address the unique treatment needs of LGBTQIA + individuals with PTSD and account for the ways that their lived experiences (e.g., discrimination, interpersonal trauma exposure) interact with their symptomatology [31, 56].

## The present study

The purpose of this scoping review is to provide an overview of the current literature on psychological interventions for LGBTQIA + individuals that target PTSD, minority stress, or both. Specifically, we aim to: (1) describe the scope of the current literature, chart available data, and synthesize findings, (2) collate information on existing PTSD and minority stress interventions for LGBTQIA + adults, and (3) identify gaps in the literature and directions for future research. Additionally, if a sufficient body of pertinent research is available, this review will also highlight promising interventions and/or key intervention components. Findings from this review will include a discussion of directions for future research and clinical implications that inform care of LGBTQIA + adults with PTSD and/or minority stress.

## Methods

This protocol follows the Preferred Reporting Items for Systematic reviews and Meta-Analyses for Scoping Reviews (PRISMA-ScR; [55]).

### Search strategy

Methods and protocol for the scoping review were pre-registered on the Open Science Framework (https://osf.io/t4fhm) using the Generalized Systematic Review Registration Form [57]. Studies for this scoping review were identified via searches of five bibliographic databases (PubMed [includes MEDLINE], EMBASE, CINAHL, PsycInfo, and Web of Science) for records with publication dates between 2000 and June 2022. This search will be replicated prior to publication for the time period between June 2022 and the present to ensure all recent relevant references are included. The searches excluded non-English language studies. The results of the database searches were uploaded to Covidence (http://covidence.org), a web-based application that facilitates screening, reviewing, and analyzing studies for knowledge syntheses such as scoping and systematic reviews. Search strings used Boolean operators to link LGBTQIA + population terms AND psychotherapy/behavioral intervention terms AND (minority stress terms OR trauma/PTSD terms) AND outcomes/study design terms. Note that these search terms will capture works focused on interventions that target PTSD only, interventions that target minority stress only, and interventions that target both PTSD and minority stress. Filters were used to restrict to English language and 2000 or later year of publication. Complete search strategies for each of the five databases, including the database's native search syntax, are available in Supplement A.

### Abstract screening and full-text review

Inclusion and exclusion criteria were developed by study team members based on study aims. Studies that were published in English and between the year 2000-present (June 2022 at time of the initial search) were included. We chose to limit our search from 2000 onward due to a paucity of research focused on minority stress prior to 2000; further, much of the terminology used in research focused on sexual and gender minorities prior to 2000 was pejorative and outdated. Our population of interest was sexual and gender minority adults. The intervention type of included studies involved any psychological, psychotherapeutic, or behavioral intervention targeting trauma, PTSD, or minority stress. To be included, any one or any combination of these outcomes of interests were included. Studies in which the intervention involved “conversion” therapy; medication only; surgical only; hormonal only were excluded. Publication type for inclusion were peer-reviewed review papers, peer-reviewed research articles, unpublished dissertations/theses, peer-reviewed clinical recommendations, and unpublished conference papers. Sources were excluded if they were letters to the editor, non-peer reviewed journals, blogs, magazines, book chapters, book reviews, retracted publications, treatment manuals, or introductions to special issues. Study designs included were case series, case studies, quality improvement, stakeholder feedback, clinical trials, and theoretical papers introducing a new treatment. Our outcomes of interest included PTSD symptoms; minority stress, including identity concealment, internalized stigma, experiences of discrimination, expectations for discrimination; resilience factors; client/patient satisfaction; implementation science outcomes (e.g., feasibility, acceptability, cost). Any studies that pertained to public health outcomes only (e.g., safe sex) were excluded. Title/abstract screening will be completed by members of the study team. Two screeners will review the title/abstract of each record to determine eligibility. Discrepancies will be resolved by a third rater (authors EAR, NM, VH). Prior to full-text review, eligibility criteria will be refined for clarity and precision. Each full-text record will be reviewed by two members of the study team. Discrepancies will be resolved by periodic discussions with the full-text review team until consensus is reached. Standardized instructions for title/abstract screening and full-text review are included in Supplement B.

### Grey literature search

Sources of grey literature include Google Scholar and ProQuest. Relevant conference proceedings will be also reviewed. These results will then be submitted to the same processes as results identified via traditional databased searches.

### Data coding, charting, and mapping

We plan to code several variables for the purposes of data charting and mapping: (1) year of publication, (2) type of work (e.g., peer-reviewed original research, clinical commentary), (3) study design, (4) population, (5) intervention targets, (6) theoretical approach of the intervention, (7) modality of the intervention, (8) intervention format, (9) professional involvement in intervention, (10) outcomes assessed, (11) measures, (12) treatment setting, and (13) findings and conclusions. See Table 1 for additional details about data coding. Further coding of variables and data mapping will be data-driven.

**Table 1:** Variables to be coded for each source

Variable	Description	Coding
Year of publication	The year the work was either published or (for unpublished works) produced	Year
Type of work	The type of work produced or published	Peer-reviewed review, peer-reviewed original research, peer-reviewed clinical commentary, peer-reviewed theoretical paper, unpublished dissertation/thesis, unpublished conference papers, other (describe)
Research design	For works that involve original research, the type of research designed used	Case study, case series, quality improvement, stakeholder feedback, pilot clinical trial, randomized control trial, other (describe)
Population	The population sampled or described	General sample including LGBTQIA + and non-LGBTQIA + individuals, broad LGBTQIA + identity, sexual minority only, gender minority only, any other subsample (specify)
Intervention Targets	For works that tested an intervention, the symptoms and/or diagnoses targeted	Trauma/PTSD only, minority stress only, both trauma/PTSD and minority stress, other (describe)
Theoretical approach	The broad theoretical approach or model underpinning the intervention. CBT approaches will be further subcoded Specific interventions will be coded via text-entry and a coding scheme will be developed empirically	Psychoanalytic/psychodynamic, supportive therapy, emotion-focused, interpersonal psychotherapy, humanistic, eclectic/integative, other (describe), cognitive behavioral (first-wave/exposure-based, second-wave/cognitive behavioral, third-wave/CBT + additional components)
Modality	The modality or service delivery method of the intervention	Traditional face-to-face, telehealth, web-based, app-based
Intervention Format	The format of the intervention	Individual, group, family, couple
Professional Involvement	Level of professional involvement in delivery of the intervention	Therapist-led, therapist-guided, peer-supported, self-guided
Outcomes Assessed	The outcomes assessed in original research studies or review papers	PTSD symptoms, minority stress (general), minority stress- internalized stigma, minority stress- concealment, minority stress- expectations of discrimination, implementation outcomes (describe), resilience, client/patient satisfaction, other mental health symptoms (describe), other behavioral health outcomes (describe), physical health outcomes (describe)
Measures	The types of measures and specific measures used to assess outcomes	Self-report measures (specify), clinical interview (specify), clinician rating scale (specify), other-report (specify), other type (specify)
Treatment Setting	Where the intervention is delivered	Mental health clinic, primary care clinic, inpatient mental health setting, inpatient medical setting, residential mental health treatment program, partial hospitalization mental health treatment program, private practice
	Was this intervention delivered at the VA	Yes or no
	Was this intervention delivered at a specialty PTSD clinic or practice	Yes or no
	Was the intervention delivered at an LGBTQIA-serving clinic or practice	Yes or no
Findings and Conclusions	What the study found and/or authors concluded	One sentence summary of findings and conclusions

### Anticipated challenges

First, this area of research is in its early stages and therefore the number of relevant works to review may be few. More specifically, we anticipate that there will be very few full-scale randomized controlled trials relevant to this research question. As such, we will be unable to perform a risk of bias assessment for this scoping review. Second, relevant works identified in this review may not provide all information we plan to code, potentially resulting in missing information for particular relevant works. To combat this, we will reach out to corresponding authors to obtain this missing information for coding purposes. However, the ability to obtain this information will depend on the responsiveness of corresponding authors. If timely responses are not provided, we will treat these coded variables as missing data. Common methodological limitations of scoping reviews also apply to our scoping review (see ref. [42]). In addition, while comprehensive, it is possible that our search strategy and grey literature search will not capture all relevant articles. In particular, restriction of works to those written in English was necessary. However, this restriction may result in important relevant works in other languages being excluded from our review.

## Discussion

To our knowledge, this review will be the first to chart the literature on interventions for both PTSD and minority stress within LGBTQIA + adults. While research on LGBTQIA + mental health is a growing field in breadth and popularity, scoping reviews targeting specific aspects of LGBTQIA + mental health are not widely available. While these areas of research are important, our review aims to narrow the scope of our search to find literature assessing specific interventions relevant to LGBTQIA + populations experiencing PTSD and minority stress.

The proposed scoping review will provide the first comprehensive overview of the state of the literature on behavioral and psychological interventions for LGBTQIA + adults targeting PTSD and/or minority stress. Results of this review will be used to identify gaps in the literature, summarize lessons learned, and inform recommendations for future research to address identified gaps. The findings of this review stand to advance the development, adaptation, and augmentation of psychological PTSD and minority stress interventions for LGBTQIA + adults. Further, if common components of effective treatments are identifiable based on available evidence, findings of this review could inform treatment development and adaptation to improve the quality of care for LGBTQIA + adults with PTSD and/or minority stress. Thus, this scoping review will be crucial in establishing the strength of the evidence-base for psychological interventions for PTSD and minority stress among LGBTQIA + adults, facilitating future research, and informing clinical care.

## Supplementary Information

Below is the link to the electronic supplementary material.

Supplementary file 1 (DOCX 2043 KB)

## Data Availability

Data and materials are available on OSF (https://osf.io/t4fhm), including pre-registration, procedures, and literature search strings. Please note that because this paper presents the protocol for an ongoing research project, additional data and materials will be uploaded as the project progresses, including a coding manual and coded data.

## Abbreviations

LGBTQIA +: Lesbian, gay, bisexual, transgender, queer, intersex, asexual, and other sexual and gender minorities
PTSD: Posttraumatic stress disorder
