# Supplementary material for: Protocol for a scoping review of PTSD and minority stress interventions for LGBTQIA + adults
Source: Discov Psychol. 2025 Jul 17;5(1):54. doi: 10.1007/s44202-025-00355-2 (PMC12270952; doi:10.1007/s44202-025-00355-2)
Supplement: Supplementary file 1 — (DOCX 2043 KB) [file 44202_2025_355_MOESM1_ESM.docx]

**SUPPLEMENT A: SEARCH STRATEGY**

**CINAHL Search String**

**PASTE THE FOLLOWING:**

(MH "sexual and gender minorities+" OR MH "transgender persons+" OR bisexual* OR F2M OR ("female-to-male" AND gender) OR gay OR gays OR "gender dysphori*" OR "gender identit*" OR "gender queer" OR genderqueer OR GLBQ* OR GLBT* OR homosexual* OR intersex OR lesbian* OR lesbigay OR LGB OR LGBT* OR M2F OR MtoF OR FtoM OR ("male-to-female" AND gender) OR queer* OR "sexual and gender minorit*" OR "sexual identit*" OR "sexual minorit*" OR "sexual orientation*" OR "sexual preference*" OR "trans fem*" OR "trans male" OR "transmasc*" OR "trans man" OR "trans men" OR "trans people" OR "trans wom*" OR transgender* OR transexual* OR "bi-umbrella" OR "trans umbrella" OR "trans and gender divers*" OR "gender divers*" OR TGNC OR "non-binary" OR nonbinary OR "gender nonconforming" OR "gender minorit*" OR BGLT* OR pansexual* OR asexual* OR omnisexual* OR "gender fluid*" OR genderfluid* OR "men loving men" OR "women loving women" OR "two spirit" OR aromantic* OR "grey sexual*" OR demisexual* OR demiromantic* OR "sexual minorit*" OR "same sex" OR "same gender" OR agender* OR sapphic OR "gender euphori*" OR panromantic* OR homoromantic* OR biromantic* OR bigender* OR "asexual umbrella" OR "gender divergen*" OR "gender atypical*" OR "gender expression*" OR butch OR femme OR "trans masc*" OR transfem* OR twink* OR "stud community" OR blaQ OR blaQueer OR "gender expansive*" OR "gender variant" OR "masculine of center" OR "feminine of center" OR "multi sexual*" "multisexual*" OR neutrois OR omnigender* OR "third gender*" OR polygender* "poly gender*" OR pangender* OR "pan gender*" OR polysexual* OR "poly sexual*" OR "romantic orientation*" OR sogie OR "sexual orientation" OR "gender identity" OR "gender expression" OR enby) AND (MH "cognitive therapy+" OR Intervention OR "cognitive behavioral therap*" OR "cognitive behavior therap*" OR "cognitive behavioural therap*" OR "cognitive behaviour therap*" OR "TF-CBT" OR "trauma-focused CBT" OR "trauma focused" OR "prolonged exposure therapy" OR "written exposure" OR "cognitive processing therapy" OR CPT OR "exposure therap*" OR psychotherap* OR "talk therap*" OR "cognitive restructur*" OR "cognitive reappraisal" OR "exposure based" OR "trauma informed" OR "evidence based" OR STAIR OR "STAIR narrative therapy" OR "narrative exposure" OR "narrative therapy" OR "peer support*" OR "therapist led" OR "therapist support*" OR "skills coach" OR "dialectical behavioral therapy" OR "dialectical behavior therapy" OR "dialectical behavioural therapy" OR "dialectical behaviour therapy" OR "cognitive therap*" OR "brief eclectic psychotherapy" OR "empirically supported" OR "trauma treatment" OR "relaxation training" OR "skills training and affective interpersonal regulation" OR EMDR OR "eye movement desensitization reprocessing" OR "eye movement desensitization and reprocessing" OR "processing therap*" OR "affirming care" OR "empowerment based" OR "LGBT affirm*" OR "LGBTQ affirm*" OR "trans affirm*" OR "LGBT-affirmative treatment" OR "behavior therap*" OR "behaviour therap*" OR "behavioral therap*" OR "behavioural therap*" OR mindfulness OR "seeking safety" OR "collaborative care" OR "stress inoculation training" OR "present centered therapy" OR "interpersonal psychotherap*" OR "acceptance and commitment therapy" OR "supportive counseling" OR "supportive therap*" OR "supportive treatment" OR "brief intervention*" OR "scalable intervention*" OR "integrated care" OR "cognitive behavioral treatment" OR "cognitive behavior treatment" OR "cognitive behavioural treatment" OR "cognitive behaviour treatment" OR "prolonged exposure treatment" OR "dialectical behavioral treatment" OR "dialectical behaviour treatment" OR "acceptance and commitment therapy" OR "acceptance and commitment treatment" OR "behavior treatment" OR "behavioral treatment" OR "behavioural treatment" OR "behaviour treatment" OR "brief eclectic treatment" OR "flooding" OR "implosive" OR "present-centered therapy" OR "cognitive restructuring" OR "trauma-related guilt reduction" OR "adapted disclosure" OR "empowering queer identities in psychotherapy" OR "effective skills to empower effective men" OR "virtual reality" OR "DBT-PE" OR "interpersonal psychotherapy" OR "accelerated resolution therapy" OR "accelerated resolution treatment" OR "imagery rehearsal therapy" OR "imagery rehearsal treatment" OR "trauma management therapy" OR "trauma management treatment" OR "processing treatment" OR "trauma therapy") AND ((MH "prejudice+" OR "minority stress" OR microaggress* OR concealment OR "concealable identit*" OR discrimination OR homophobia OR biphobia OR transphobia OR harassment OR victimization OR heteronorm* OR homonorm* OR "hate crim*" OR prejudice* OR systemic* OR barriers OR "expectations of rejection" OR "expectation of rejection" OR tokenism OR tokeniz* OR minoritiz* OR marginaliz* OR bias OR burden OR "micro-aggress*" OR macroaggress* OR "macro-aggress*" OR rejection OR lesbophobia OR heterosexis* OR cissexis* OR stigma* OR "traumatic invalidation" OR "family rejection" OR oppression OR oppressive OR outness OR stereotyp*) OR (PTSD OR posttrauma* OR trauma* OR "Criterion A stressor*" OR PTS OR avoidance OR "traumatic intrusion*" OR "intrusive thought*" OR "intrusive cognition*" OR "intrusive memor*" OR hyperarousal OR hypervigilenc* OR "post-trauma*" OR "traumatic injury" OR "acute stress disorder*" OR "moral injur*" OR reexperiencing OR "re-experiencing" OR "negative alterations in cognit*" OR "negative alteration in mood" OR "fear structure*")) AND (effective* OR efficac* OR feasibility OR acceptability OR implementation OR sustainability OR adoption OR appropriateness OR fidelity OR "implementation cost*" OR "patient experience*" OR "symptom reduction*" OR applicability OR "quality improvement" OR "program evaluation" OR "provider perspective*" OR "provider interview*" OR "stakeholder perspective*" OR "stakeholder interview*" OR "stakeholder feedback" OR "provider feedback" OR "end-user input" OR "symptom change*" OR "PCL-5" OR "PTSD checklist" OR "randomized control trial" OR "randomized controlled trial*" OR RCT OR "pilot trial" OR "pilot study" OR review OR "treatment recommendation*" OR "clinical recommendation*" OR "treatment consideration*" OR "treatment recommendation*" OR "case series" OR "case stud*" OR "clinical trial*" OR "participatory research" OR "comparative effectiveness" OR "comparative efficacy" OR "case conceptualization*" OR "case formulation*" OR qualitative OR "participatory design" OR "human-centered design" OR "human centered design" OR "community engage*" OR "focus group*" OR "focus-group*" OR "clinical commentar*" OR editorial* OR "clinical consideration*")

**1: Population terms**

(MH "sexual and gender minorities+" OR MH "transgender persons+" OR bisexual* OR F2M OR ("female-to-male" AND gender) OR gay OR gays OR "gender dysphori*" OR "gender identit*" OR "gender queer" OR genderqueer OR GLBQ* OR GLBT* OR homosexual* OR intersex OR lesbian* OR lesbigay OR LGB OR LGBT* OR M2F OR MtoF OR FtoM OR ("male-to-female" AND gender) OR queer* OR "sexual and gender minorit*" OR "sexual identit*" OR "sexual minorit*" OR "sexual orientation*" OR "sexual preference*" OR "trans fem*" OR "trans male" OR "transmasc*" OR "trans man" OR "trans men" OR "trans people" OR "trans wom*" OR transgender* OR transexual* OR "bi-umbrella" OR "trans umbrella" OR "trans and gender divers*" OR "gender divers*" OR TGNC OR "non-binary" OR nonbinary OR "gender nonconforming" OR "gender minorit*" OR BGLT* OR pansexual* OR asexual* OR omnisexual* OR "gender fluid*" OR genderfluid* OR "men loving men" OR "women loving women" OR "two spirit" OR aromantic* OR "grey sexual*" OR demisexual* OR demiromantic* OR "sexual minorit*" OR "same sex" OR "same gender" OR agender* OR sapphic OR "gender euphori*" OR panromantic* OR homoromantic* OR biromantic* OR bigender* OR "asexual umbrella" OR "gender divergen*" OR "gender atypical*" OR "gender expression*" OR butch OR femme OR "trans masc*" OR transfem* OR twink* OR "stud community" OR blaQ OR blaQueer OR "gender expansive*" OR "gender variant" OR "masculine of center" OR "feminine of center" OR "multi sexual*" "multisexual*" OR neutrois OR omnigender* OR "third gender*" OR polygender* "poly gender*" OR pangender* OR "pan gender*" OR polysexual* OR "poly sexual*" OR "romantic orientation*" OR sogie OR "sexual orientation" OR "gender identity" OR "gender expression" OR enby)

**2: Treatment Terms**

(MH "cognitive therapy+" OR Intervention OR "cognitive behavioral therap*" OR "cognitive behavior therap*" OR "cognitive behavioural therap*" OR "cognitive behaviour therap*" OR "TF-CBT" OR "trauma-focused CBT" OR "trauma focused" OR "prolonged exposure therapy" OR "written exposure" OR "cognitive processing therapy" OR CPT OR "exposure therap*" OR psychotherap* OR "talk therap*" OR "cognitive restructur*" OR "cognitive reappraisal" OR "exposure based" OR "trauma informed" OR "evidence based" OR STAIR OR "STAIR narrative therapy" OR "narrative exposure" OR "narrative therapy" OR "peer support*" OR "therapist led" OR "therapist support*" OR "skills coach" OR "dialectical behavioral therapy" OR "dialectical behavior therapy" OR "dialectical behavioural therapy" OR "dialectical behaviour therapy" OR "cognitive therap*" OR "brief eclectic psychotherapy" OR "empirically supported" OR "trauma treatment" OR "relaxation training" OR "skills training and affective interpersonal regulation" OR EMDR OR "eye movement desensitization reprocessing" OR "eye movement desensitization and reprocessing" OR "processing therap*" OR "affirming care" OR "empowerment based" OR "LGBT affirm*" OR "LGBTQ affirm*" OR "trans affirm*" OR "LGBT-affirmative treatment" OR "behavior therap*" OR "behaviour therap*" OR "behavioral therap*" OR "behavioural therap*" OR mindfulness OR "seeking safety" OR "collaborative care" OR "stress inoculation training" OR "present centered therapy" OR "interpersonal psychotherap*" OR "acceptance and commitment therapy" OR "supportive counseling" OR "supportive therap*" OR "supportive treatment" OR "brief intervention*" OR "scalable intervention*" OR "integrated care" OR "cognitive behavioral treatment" OR "cognitive behavior treatment" OR "cognitive behavioural treatment" OR "cognitive behaviour treatment" OR "prolonged exposure treatment" OR "dialectical behavioral treatment" OR "dialectical behaviour treatment" OR "acceptance and commitment therapy" OR "acceptance and commitment treatment" OR "behavior treatment" OR "behavioral treatment" OR "behavioural treatment" OR "behaviour treatment" OR "brief eclectic treatment" OR "flooding" OR "implosive" OR "present-centered therapy" OR "cognitive restructuring" OR "trauma-related guilt reduction" OR "adapted disclosure" OR "empowering queer identities in psychotherapy" OR "effective skills to empower effective men" OR "virtual reality" OR "DBT-PE" OR "interpersonal psychotherapy" OR "accelerated resolution therapy" OR "accelerated resolution treatment" OR "imagery rehearsal therapy" OR "imagery rehearsal treatment" OR "trauma management therapy" OR "trauma management treatment" OR "processing treatment" OR "trauma therapy")

**3: Minority Stress Terms**

(MH "prejudice+" OR "minority stress" OR microaggress* OR concealment OR "concealable identit*" OR discrimination OR homophobia OR biphobia OR transphobia OR harassment OR victimization OR heteronorm* OR homonorm* OR "hate crim*" OR prejudice* OR systemic* OR barriers OR "expectations of rejection" OR "expectation of rejection" OR tokenism OR tokeniz* OR minoritiz* OR marginaliz* OR bias OR burden OR "micro-aggress*" OR macroaggress* OR "macro-aggress*" OR rejection OR lesbophobia OR heterosexis* OR cissexis* OR stigma* OR "traumatic invalidation" OR "family rejection" OR oppression OR oppressive OR outness OR stereotyp*)

**4: Trauma Terms**

(PTSD OR posttrauma* OR trauma* OR "Criterion A stressor*" OR PTS OR avoidance OR "traumatic intrusion*" OR "intrusive thought*" OR "intrusive cognition*" OR "intrusive memor*" OR hyperarousal OR hypervigilenc* OR "post-trauma*" OR "traumatic injury" OR "acute stress disorder*" OR "moral injur*" OR reexperiencing OR "re-experiencing" OR "negative alterations in cognit*" OR "negative alteration in mood" OR "fear structure*")

**5. Outcome & Design Terms**

("treatment outcome*" OR "program evaluation" OR effective* OR efficac* OR feasibility OR acceptability OR implementation OR sustainability OR adoption OR appropriateness OR fidelity OR "implementation cost*" OR "patient experience*" OR "symptom reduction*" OR applicability OR "quality improvement" OR "program evaluation" OR "provider perspective*" OR "provider interview*" OR "stakeholder perspective*" OR "stakeholder interview*" OR "stakeholder feedback" OR "provider feedback" OR "end-user input" OR "symptom change*" OR "PCL-5" OR "PTSD checklist" OR "randomized control trial" OR "randomized controlled trial*" OR RCT OR "pilot trial" OR "pilot study" OR review OR "treatment recommendation*" OR "clinical recommendation*" OR "treatment consideration*" OR "treatment recommendation*" OR "case series" OR "case stud*" OR "clinical trial*" OR "participatory research" OR "comparative effectiveness" OR "comparative efficacy" OR "case conceptualization*" OR "case formulation*" OR qualitative OR "participatory design" OR "human-centered design" OR "human centered design" OR "community engage*" OR "focus group*" OR "focus-group*" OR "clinical commentar*" OR editorial* OR "clinical consideration*")

**Embase Search Strategy**

**Embase search #1 imported into covidence + email alert set up on 9/22/2022**

**1: Population terms**

('transsexualism'/exp OR 'sexual and gender minority'/exp OR 'bisexuality'/exp OR 'homosexuality'/exp OR 'transgender'/exp OR bisexual*:ti,ab,kw,de OR F2M:ti,ab,kw,de OR ("female-to-male":ti,ab,kw,de AND gender:ti,ab,kw,de) OR gay:ti,ab,kw,de OR gays:ti,ab,kw,de OR "gender dysphori*":ti,ab,kw,de OR "gender identit*":ti,ab,kw,de OR "gender queer":ti,ab,kw,de OR genderqueer:ti,ab,kw,de OR GLBQ*:ti,ab,kw,de OR GLBT*:ti,ab,kw,de OR homosexual*:ti,ab,kw,de OR intersex:ti,ab,kw,de OR lesbian*:ti,ab,kw,de OR lesbigay:ti,ab,kw,de OR LGB:ti,ab,kw,de OR LGBT*:ti,ab,kw,de OR M2F:ti,ab,kw,de OR MtoF:ti,ab,kw,de OR FtoM:ti,ab,kw,de OR ("male-to-female":ti,ab,kw,de AND gender) OR queer*:ti,ab,kw,de OR "sexual and gender minorit*":ti,ab,kw,de OR "sexual identit*":ti,ab,kw,de OR "sexual minorit*":ti,ab,kw,de OR "sexual orientation*":ti,ab,kw,de OR "sexual preference*":ti,ab,kw,de OR "trans fem*":ti,ab,kw,de OR "trans male":ti,ab,kw,de OR "transmasc*" OR "trans man":ti,ab,kw,de OR "trans men":ti,ab,kw,de OR "trans people":ti,ab,kw,de OR "trans wom*":ti,ab,kw,de OR transgender*:ti,ab,kw,de OR transexual*:ti,ab,kw,de OR "bi-umbrella":ti,ab,kw,de OR "trans umbrella":ti,ab,kw,de OR "trans and gender divers*":ti,ab,kw,de OR "gender divers*":ti,ab,kw,de OR TGNC:ti,ab,kw,de OR "non-binary":ti,ab,kw,de OR nonbinary:ti,ab,kw,de OR "gender nonconforming":ti,ab,kw,de OR "gender minorit*":ti,ab,kw,de OR BGLT*:ti,ab,kw,de OR pansexual*:ti,ab,kw,de OR asexual*:ti,ab,kw,de OR omnisexual*:ti,ab,kw,de OR "gender fluid*":ti,ab,kw,de OR genderfluid*:ti,ab,kw,de OR "men loving men":ti,ab,kw,de OR "women loving women":ti,ab,kw,de OR "two spirit":ti,ab,kw,de OR aromantic*:ti,ab,kw,de OR "grey sexual*":ti,ab,kw,de OR demisexual*:ti,ab,kw,de OR demiromantic*:ti,ab,kw,de OR "sexual minorit*":ti,ab,kw,de OR "same sex":ti,ab,kw,de OR "same gender":ti,ab,kw,de OR agender*:ti,ab,kw,de OR sapphic:ti,ab,kw,de OR "gender euphori*":ti,ab,kw,de OR panromantic*:ti,ab,kw,de OR homoromantic*:ti,ab,kw,de OR biromantic*:ti,ab,kw,de OR bigender*:ti,ab,kw,de OR "asexual umbrella":ti,ab,kw,de OR "gender divergen*":ti,ab,kw,de OR "gender atypical*":ti,ab,kw,de OR "gender expression*":ti,ab,kw,de OR butch:ti,ab,kw,de OR femme:ti,ab,kw,de OR "trans masc*":ti,ab,kw,de OR transfem*:ti,ab,kw,de OR twink*:ti,ab,kw,de OR "stud community":ti,ab,kw,de OR blaQ:ti,ab,kw,de OR blaQueer:ti,ab,kw,de OR "gender expansive*":ti,ab,kw,de OR "gender variant":ti,ab,kw,de OR "masculine of center":ti,ab,kw,de OR "feminine of center":ti,ab,kw,de OR "multi sexual*":ti,ab,kw,de "multisexual*":ti,ab,kw,de OR neutrois:ti,ab,kw,de OR omnigender*:ti,ab,kw,de OR "third gender*":ti,ab,kw,de OR polygender*:ti,ab,kw,de "poly gender*":ti,ab,kw,de OR pangender*:ti,ab,kw,de OR "pan gender*":ti,ab,kw,de OR polysexual*:ti,ab,kw,de OR "poly sexual*":ti,ab,kw,de OR "romantic orientation*":ti,ab,kw,de OR sogie:ti,ab,kw,de OR "sexual orientation":ti,ab,kw,de OR "gender identity":ti,ab,kw,de OR "gender expression":ti,ab,kw,de OR enby:ti,ab,kw,de)

**2: Treatment Terms**

('psychotherapy'/exp OR 'cognitive behavioral therapy'/exp OR Intervention:ti,ab,kw,de OR "cognitive behavioral therap*":ti,ab,kw,de OR "cognitive behavior therap*":ti,ab,kw,de OR "cognitive behavioural therap*":ti,ab,kw,de OR "cognitive behaviour therap*":ti,ab,kw,de OR "TF-CBT":ti,ab,kw,de OR "trauma-focused CBT":ti,ab,kw,de OR "trauma focused":ti,ab,kw,de OR "prolonged exposure therapy":ti,ab,kw,de OR "written exposure":ti,ab,kw,de OR "cognitive processing therapy":ti,ab,kw,de OR CPT:ti,ab,kw,de OR "exposure therap*":ti,ab,kw,de OR psychotherap*:ti,ab,kw,de OR "talk therap*":ti,ab,kw,de OR "cognitive restructur*":ti,ab,kw,de OR "cognitive reappraisal":ti,ab,kw,de OR "exposure based":ti,ab,kw,de OR "trauma informed":ti,ab,kw,de OR "evidence based":ti,ab,kw,de OR STAIR:ti,ab,kw,de OR "STAIR narrative therapy":ti,ab,kw,de OR "narrative exposure":ti,ab,kw,de OR "narrative therapy":ti,ab,kw,de OR "peer support*":ti,ab,kw,de OR "therapist led":ti,ab,kw,de OR "therapist support*":ti,ab,kw,de OR "skills coach":ti,ab,kw,de OR "dialectical behavioral therapy":ti,ab,kw,de OR "dialectical behavior therapy":ti,ab,kw,de OR "dialectical behavioural therapy":ti,ab,kw,de OR "dialectical behaviour therapy":ti,ab,kw,de OR "cognitive therap*":ti,ab,kw,de OR "brief eclectic psychotherapy":ti,ab,kw,de OR "empirically supported":ti,ab,kw,de OR "trauma treatment":ti,ab,kw,de OR "relaxation training":ti,ab,kw,de OR "skills training and affective interpersonal regulation":ti,ab,kw,de OR EMDR:ti,ab,kw,de OR "eye movement desensitization reprocessing":ti,ab,kw,de OR "eye movement desensitization and reprocessing":ti,ab,kw,de OR "processing therap*":ti,ab,kw,de OR "affirming care":ti,ab,kw,de OR "empowerment based":ti,ab,kw,de OR "LGBT affirm*":ti,ab,kw,de OR "LGBTQ affirm*":ti,ab,kw,de OR "trans affirm*":ti,ab,kw,de OR "LGBT-affirmative treatment":ti,ab,kw,de OR "behavior therap*":ti,ab,kw,de OR "behaviour therap*":ti,ab,kw,de OR "behavioral therap*":ti,ab,kw,de OR "behavioural therap*":ti,ab,kw,de OR mindfulness:ti,ab,kw,de OR "seeking safety":ti,ab,kw,de OR "collaborative care":ti,ab,kw,de OR "stress inoculation training":ti,ab,kw,de OR "present centered therapy":ti,ab,kw,de OR "interpersonal psychotherap*":ti,ab,kw,de OR "acceptance and commitment therapy":ti,ab,kw,de OR "supportive counseling":ti,ab,kw,de OR "supportive therap*":ti,ab,kw,de OR "supportive treatment":ti,ab,kw,de OR "brief intervention*":ti,ab,kw,de OR "scalable intervention*":ti,ab,kw,de OR "integrated care":ti,ab,kw,de OR "cognitive behavioral treatment":ti,ab,kw,de OR "cognitive behavior treatment":ti,ab,kw,de OR "cognitive behavioural treatment":ti,ab,kw,de OR "cognitive behaviour treatment":ti,ab,kw,de OR "prolonged exposure treatment":ti,ab,kw,de OR "dialectical behavioral treatment":ti,ab,kw,de OR "dialectical behaviour treatment":ti,ab,kw,de OR "acceptance and commitment therapy":ti,ab,kw,de OR "acceptance and commitment treatment":ti,ab,kw,de OR "behavior treatment":ti,ab,kw,de OR "behavioral treatment":ti,ab,kw,de OR "behavioural treatment":ti,ab,kw,de OR "behaviour treatment":ti,ab,kw,de OR "brief eclectic treatment":ti,ab,kw,de OR "flooding":ti,ab,kw,de OR "implosive":ti,ab,kw,de OR "present-centered therapy":ti,ab,kw,de OR "cognitive restructuring":ti,ab,kw,de OR "trauma-related guilt reduction":ti,ab,kw,de OR "adapted disclosure":ti,ab,kw,de OR "empowering queer identities in psychotherapy":ti,ab,kw,de OR "effective skills to empower effective men":ti,ab,kw,de OR "virtual reality":ti,ab,kw,de OR "DBT-PE":ti,ab,kw,de OR "interpersonal psychotherapy":ti,ab,kw,de OR "accelerated resolution therapy":ti,ab,kw,de OR "accelerated resolution treatment":ti,ab,kw,de OR "imagery rehearsal therapy":ti,ab,kw,de OR "imagery rehearsal treatment":ti,ab,kw,de OR "trauma management therapy":ti,ab,kw,de OR "trauma management treatment":ti,ab,kw,de OR "processing treatment":ti,ab,kw,de OR "trauma therapy":ti,ab,kw,de)

**3: Minority Stress Terms**

('microaggression'/exp OR 'prejudice'/exp OR "minority stress":ti,ab,kw,de OR microaggress*:ti,ab,kw,de OR concealment:ti,ab,kw,de OR "concealable identit*":ti,ab,kw,de OR discrimination:ti,ab,kw,de OR homophobia:ti,ab,kw,de OR biphobia:ti,ab,kw,de OR transphobia:ti,ab,kw,de OR harassment:ti,ab,kw,de OR victimization:ti,ab,kw,de OR heteronorm*:ti,ab,kw,de OR homonorm*:ti,ab,kw,de OR "hate crim*":ti,ab,kw,de OR prejudice*:ti,ab,kw,de OR systemic*:ti,ab,kw,de OR barriers:ti,ab,kw,de OR "expectations of rejection":ti,ab,kw,de OR "expectation of rejection":ti,ab,kw,de OR tokenism:ti,ab,kw,de tokeniz*:ti,ab,kw,de OR minoritiz*:ti,ab,kw,de OR marginaliz*:ti,ab,kw,de OR bias:ti,ab,kw,de OR burden:ti,ab,kw,de OR "micro-aggress*":ti,ab,kw,de OR macroaggress*:ti,ab,kw,de OR "macro-aggress*":ti,ab,kw,de OR rejection:ti,ab,kw,de OR lesbophobia:ti,ab,kw,de OR heterosexis*:ti,ab,kw,de OR cissexis*:ti,ab,kw,de OR stigma*:ti,ab,kw,de OR "traumatic invalidation":ti,ab,kw,de OR "family rejection":ti,ab,kw,de OR oppression:ti,ab,kw,de OR oppressive:ti,ab,kw,de OR outness:ti,ab,kw,de OR stereotyp*:ti,ab,kw,de)

**4: Trauma Terms**

('anxiety disorder'/exp OR PTSD:ti,ab,kw,de OR posttrauma*:ti,ab,kw,de OR trauma*:ti,ab,kw,de OR "Criterion A stressor*":ti,ab,kw,de OR PTS:ti,ab,kw,de OR avoidance:ti,ab,kw,de OR "traumatic intrusion*":ti,ab,kw,de OR "intrusive thought*":ti,ab,kw,de OR "intrusive cognition*":ti,ab,kw,de OR "intrusive memor*":ti,ab,kw,de OR hyperarousal:ti,ab,kw,de OR hypervigilenc*:ti,ab,kw,de OR "post-trauma*":ti,ab,kw,de OR "traumatic injury":ti,ab,kw,de OR "acute stress disorder*":ti,ab,kw,de OR "moral injur*":ti,ab,kw,de OR reexperiencing:ti,ab,kw,de OR "re-experiencing":ti,ab,kw,de OR "negative alterations in cognit*":ti,ab,kw,de OR "negative alteration in mood":ti,ab,kw,de OR "fear structure*":ti,ab,kw,de)

**5. Outcome & Design Terms**

('stakeholder engagement'/exp OR 'treatment outcome'/exp OR 'total quality management'/exp OR 'program evaluation'/exp OR 'implementation science'/exp OR effective*:ti,ab,kw,de OR efficac*:ti,ab,kw,de OR feasibility:ti,ab,kw,de OR acceptability:ti,ab,kw,de OR implementation:ti,ab,kw,de OR sustainability:ti,ab,kw,de OR adoption:ti,ab,kw,de OR appropriateness:ti,ab,kw,de OR fidelity:ti,ab,kw,de OR "implementation cost*":ti,ab,kw,de OR "patient experience*":ti,ab,kw,de OR "symptom reduction*":ti,ab,kw,de OR applicability:ti,ab,kw,de OR "quality improvement":ti,ab,kw,de OR "program evaluation":ti,ab,kw,de OR "provider perspective*":ti,ab,kw,de OR "provider interview*":ti,ab,kw,de OR "stakeholder perspective*":ti,ab,kw,de OR "stakeholder interview*":ti,ab,kw,de OR "stakeholder feedback":ti,ab,kw,de OR "provider feedback":ti,ab,kw,de OR "end-user input":ti,ab,kw,de OR "symptom change*":ti,ab,kw,de OR "PCL-5":ti,ab,kw,de OR "PTSD checklist":ti,ab,kw,de OR "randomized control trial":ti,ab,kw,de OR "randomized controlled trial*":ti,ab,kw,de OR RCT:ti,ab,kw,de OR "pilot trial":ti,ab,kw,de OR "pilot study":ti,ab,kw,de OR review:ti,ab,kw,de OR "treatment recommendation*":ti,ab,kw,de OR "clinical recommendation*":ti,ab,kw,de OR "treatment consideration*":ti,ab,kw,de OR "treatment recommendation*":ti,ab,kw,de OR "case series":ti,ab,kw,de OR "case stud*":ti,ab,kw,de OR "clinical trial*":ti,ab,kw,de OR "participatory research":ti,ab,kw,de OR "comparative effectiveness":ti,ab,kw,de OR "comparative efficacy":ti,ab,kw,de OR "case conceptualization*":ti,ab,kw,de OR "case formulation*":ti,ab,kw,de OR qualitative:ti,ab,kw,de OR "participatory design":ti,ab,kw,de OR "human-centered design":ti,ab,kw,de OR "human centered design":ti,ab,kw,de OR "community engage*":ti,ab,kw,de OR "focus group*":ti,ab,kw,de OR "focus-group*":ti,ab,kw,de OR "clinical commentar*":ti,ab,kw,de OR editorial*:ti,ab,kw,de OR "clinical consideration*":ti,ab,kw,de)

**6. Limits to date/language/Embase-only (tries to exclude most if not all of the PubMed duplicates)**

AND [embase]/lim NOT ([embase]/lim AND [medline]/lim)

**PASTE INTO EMBASE:**

('transsexualism'/exp OR 'sexual and gender minority'/exp OR 'bisexuality'/exp OR 'homosexuality'/exp OR 'transgender'/exp OR bisexual*:ti,ab,kw,de OR F2M:ti,ab,kw,de OR ("female-to-male":ti,ab,kw,de AND gender:ti,ab,kw,de) OR gay:ti,ab,kw,de OR gays:ti,ab,kw,de OR "gender dysphori*":ti,ab,kw,de OR "gender identit*":ti,ab,kw,de OR "gender queer":ti,ab,kw,de OR genderqueer:ti,ab,kw,de OR GLBQ*:ti,ab,kw,de OR GLBT*:ti,ab,kw,de OR homosexual*:ti,ab,kw,de OR intersex:ti,ab,kw,de OR lesbian*:ti,ab,kw,de OR lesbigay:ti,ab,kw,de OR LGB:ti,ab,kw,de OR LGBT*:ti,ab,kw,de OR M2F:ti,ab,kw,de OR MtoF:ti,ab,kw,de OR FtoM:ti,ab,kw,de OR ("male-to-female":ti,ab,kw,de AND gender) OR queer*:ti,ab,kw,de OR "sexual and gender minorit*":ti,ab,kw,de OR "sexual identit*":ti,ab,kw,de OR "sexual minorit*":ti,ab,kw,de OR "sexual orientation*":ti,ab,kw,de OR "sexual preference*":ti,ab,kw,de OR "trans fem*":ti,ab,kw,de OR "trans male":ti,ab,kw,de OR "transmasc*" OR "trans man":ti,ab,kw,de OR "trans men":ti,ab,kw,de OR "trans people":ti,ab,kw,de OR "trans wom*":ti,ab,kw,de OR transgender*:ti,ab,kw,de OR transexual*:ti,ab,kw,de OR "bi-umbrella":ti,ab,kw,de OR "trans umbrella":ti,ab,kw,de OR "trans and gender divers*":ti,ab,kw,de OR "gender divers*":ti,ab,kw,de OR TGNC:ti,ab,kw,de OR "non-binary":ti,ab,kw,de OR nonbinary:ti,ab,kw,de OR "gender nonconforming":ti,ab,kw,de OR "gender minorit*":ti,ab,kw,de OR BGLT*:ti,ab,kw,de OR pansexual*:ti,ab,kw,de OR asexual*:ti,ab,kw,de OR omnisexual*:ti,ab,kw,de OR "gender fluid*":ti,ab,kw,de OR genderfluid*:ti,ab,kw,de OR "men loving men":ti,ab,kw,de OR "women loving women":ti,ab,kw,de OR "two spirit":ti,ab,kw,de OR aromantic*:ti,ab,kw,de OR "grey sexual*":ti,ab,kw,de OR demisexual*:ti,ab,kw,de OR demiromantic*:ti,ab,kw,de OR "sexual minorit*":ti,ab,kw,de OR "same sex":ti,ab,kw,de OR "same gender":ti,ab,kw,de OR agender*:ti,ab,kw,de OR sapphic:ti,ab,kw,de OR "gender euphori*":ti,ab,kw,de OR panromantic*:ti,ab,kw,de OR homoromantic*:ti,ab,kw,de OR biromantic*:ti,ab,kw,de OR bigender*:ti,ab,kw,de OR "asexual umbrella":ti,ab,kw,de OR "gender divergen*":ti,ab,kw,de OR "gender atypical*":ti,ab,kw,de OR "gender expression*":ti,ab,kw,de OR butch:ti,ab,kw,de OR femme:ti,ab,kw,de OR "trans masc*":ti,ab,kw,de OR transfem*:ti,ab,kw,de OR twink*:ti,ab,kw,de OR "stud community":ti,ab,kw,de OR blaQ:ti,ab,kw,de OR blaQueer:ti,ab,kw,de OR "gender expansive*":ti,ab,kw,de OR "gender variant":ti,ab,kw,de OR "masculine of center":ti,ab,kw,de OR "feminine of center":ti,ab,kw,de OR "multi sexual*":ti,ab,kw,de "multisexual*":ti,ab,kw,de OR neutrois:ti,ab,kw,de OR omnigender*:ti,ab,kw,de OR "third gender*":ti,ab,kw,de OR polygender*:ti,ab,kw,de "poly gender*":ti,ab,kw,de OR pangender*:ti,ab,kw,de OR "pan gender*":ti,ab,kw,de OR polysexual*:ti,ab,kw,de OR "poly sexual*":ti,ab,kw,de OR "romantic orientation*":ti,ab,kw,de OR sogie:ti,ab,kw,de OR "sexual orientation":ti,ab,kw,de OR "gender identity":ti,ab,kw,de OR "gender expression":ti,ab,kw,de OR enby:ti,ab,kw,de) AND ('psychotherapy'/exp OR 'cognitive behavioral therapy'/exp OR Intervention:ti,ab,kw,de OR "cognitive behavioral therap*":ti,ab,kw,de OR "cognitive behavior therap*":ti,ab,kw,de OR "cognitive behavioural therap*":ti,ab,kw,de OR "cognitive behaviour therap*":ti,ab,kw,de OR "TF-CBT":ti,ab,kw,de OR "trauma-focused CBT":ti,ab,kw,de OR "trauma focused":ti,ab,kw,de OR "prolonged exposure therapy":ti,ab,kw,de OR "written exposure":ti,ab,kw,de OR "cognitive processing therapy":ti,ab,kw,de OR CPT:ti,ab,kw,de OR "exposure therap*":ti,ab,kw,de OR psychotherap*:ti,ab,kw,de OR "talk therap*":ti,ab,kw,de OR "cognitive restructur*":ti,ab,kw,de OR "cognitive reappraisal":ti,ab,kw,de OR "exposure based":ti,ab,kw,de OR "trauma informed":ti,ab,kw,de OR "evidence based":ti,ab,kw,de OR STAIR:ti,ab,kw,de OR "STAIR narrative therapy":ti,ab,kw,de OR "narrative exposure":ti,ab,kw,de OR "narrative therapy":ti,ab,kw,de OR "peer support*":ti,ab,kw,de OR "therapist led":ti,ab,kw,de OR "therapist support*":ti,ab,kw,de OR "skills coach":ti,ab,kw,de OR "dialectical behavioral therapy":ti,ab,kw,de OR "dialectical behavior therapy":ti,ab,kw,de OR "dialectical behavioural therapy":ti,ab,kw,de OR "dialectical behaviour therapy":ti,ab,kw,de OR "cognitive therap*":ti,ab,kw,de OR "brief eclectic psychotherapy":ti,ab,kw,de OR "empirically supported":ti,ab,kw,de OR "trauma treatment":ti,ab,kw,de OR "relaxation training":ti,ab,kw,de OR "skills training and affective interpersonal regulation":ti,ab,kw,de OR EMDR:ti,ab,kw,de OR "eye movement desensitization reprocessing":ti,ab,kw,de OR "eye movement desensitization and reprocessing":ti,ab,kw,de OR "processing therap*":ti,ab,kw,de OR "affirming care":ti,ab,kw,de OR "empowerment based":ti,ab,kw,de OR "LGBT affirm*":ti,ab,kw,de OR "LGBTQ affirm*":ti,ab,kw,de OR "trans affirm*":ti,ab,kw,de OR "LGBT-affirmative treatment":ti,ab,kw,de OR "behavior therap*":ti,ab,kw,de OR "behaviour therap*":ti,ab,kw,de OR "behavioral therap*":ti,ab,kw,de OR "behavioural therap*":ti,ab,kw,de OR mindfulness:ti,ab,kw,de OR "seeking safety":ti,ab,kw,de OR "collaborative care":ti,ab,kw,de OR "stress inoculation training":ti,ab,kw,de OR "present centered therapy":ti,ab,kw,de OR "interpersonal psychotherap*":ti,ab,kw,de OR "acceptance and commitment therapy":ti,ab,kw,de OR "supportive counseling":ti,ab,kw,de OR "supportive therap*":ti,ab,kw,de OR "supportive treatment":ti,ab,kw,de OR "brief intervention*":ti,ab,kw,de OR "scalable intervention*":ti,ab,kw,de OR "integrated care":ti,ab,kw,de OR "cognitive behavioral treatment":ti,ab,kw,de OR "cognitive behavior treatment":ti,ab,kw,de OR "cognitive behavioural treatment":ti,ab,kw,de OR "cognitive behaviour treatment":ti,ab,kw,de OR "prolonged exposure treatment":ti,ab,kw,de OR "dialectical behavioral treatment":ti,ab,kw,de OR "dialectical behaviour treatment":ti,ab,kw,de OR "acceptance and commitment therapy":ti,ab,kw,de OR "acceptance and commitment treatment":ti,ab,kw,de OR "behavior treatment":ti,ab,kw,de OR "behavioral treatment":ti,ab,kw,de OR "behavioural treatment":ti,ab,kw,de OR "behaviour treatment":ti,ab,kw,de OR "brief eclectic treatment":ti,ab,kw,de OR "flooding":ti,ab,kw,de OR "implosive":ti,ab,kw,de OR "present-centered therapy":ti,ab,kw,de OR "cognitive restructuring":ti,ab,kw,de OR "trauma-related guilt reduction":ti,ab,kw,de OR "adapted disclosure":ti,ab,kw,de OR "empowering queer identities in psychotherapy":ti,ab,kw,de OR "effective skills to empower effective men":ti,ab,kw,de OR "virtual reality":ti,ab,kw,de OR "DBT-PE":ti,ab,kw,de OR "interpersonal psychotherapy":ti,ab,kw,de OR "accelerated resolution therapy":ti,ab,kw,de OR "accelerated resolution treatment":ti,ab,kw,de OR "imagery rehearsal therapy":ti,ab,kw,de OR "imagery rehearsal treatment":ti,ab,kw,de OR "trauma management therapy":ti,ab,kw,de OR "trauma management treatment":ti,ab,kw,de OR "processing treatment":ti,ab,kw,de OR "trauma therapy":ti,ab,kw,de) AND (('anxiety disorder'/exp OR PTSD:ti,ab,kw,de OR posttrauma*:ti,ab,kw,de OR trauma*:ti,ab,kw,de OR "Criterion A stressor*":ti,ab,kw,de OR PTS:ti,ab,kw,de OR avoidance:ti,ab,kw,de OR "traumatic intrusion*":ti,ab,kw,de OR "intrusive thought*":ti,ab,kw,de OR "intrusive cognition*":ti,ab,kw,de OR "intrusive memor*":ti,ab,kw,de OR hyperarousal:ti,ab,kw,de OR hypervigilenc*:ti,ab,kw,de OR "post-trauma*":ti,ab,kw,de OR "traumatic injury":ti,ab,kw,de OR "acute stress disorder*":ti,ab,kw,de OR "moral injur*":ti,ab,kw,de OR reexperiencing:ti,ab,kw,de OR "re-experiencing":ti,ab,kw,de OR "negative alterations in cognit*":ti,ab,kw,de OR "negative alteration in mood":ti,ab,kw,de OR "fear structure*":ti,ab,kw,de) OR ('microaggression'/exp OR 'prejudice'/exp OR "minority stress":ti,ab,kw,de OR microaggress*:ti,ab,kw,de OR concealment:ti,ab,kw,de OR "concealable identit*":ti,ab,kw,de OR discrimination:ti,ab,kw,de OR homophobia:ti,ab,kw,de OR biphobia:ti,ab,kw,de OR transphobia:ti,ab,kw,de OR harassment:ti,ab,kw,de OR victimization:ti,ab,kw,de OR heteronorm*:ti,ab,kw,de OR homonorm*:ti,ab,kw,de OR "hate crim*":ti,ab,kw,de OR prejudice*:ti,ab,kw,de OR systemic*:ti,ab,kw,de OR barriers:ti,ab,kw,de OR "expectations of rejection":ti,ab,kw,de OR "expectation of rejection":ti,ab,kw,de OR tokenism:ti,ab,kw,de tokeniz*:ti,ab,kw,de OR minoritiz*:ti,ab,kw,de OR marginaliz*:ti,ab,kw,de OR bias:ti,ab,kw,de OR burden:ti,ab,kw,de OR "micro-aggress*":ti,ab,kw,de OR macroaggress*:ti,ab,kw,de OR "macro-aggress*":ti,ab,kw,de OR rejection:ti,ab,kw,de OR lesbophobia:ti,ab,kw,de OR heterosexis*:ti,ab,kw,de OR cissexis*:ti,ab,kw,de OR stigma*:ti,ab,kw,de OR "traumatic invalidation":ti,ab,kw,de OR "family rejection":ti,ab,kw,de OR oppression:ti,ab,kw,de OR oppressive:ti,ab,kw,de OR outness:ti,ab,kw,de OR stereotyp*:ti,ab,kw,de)) AND ('stakeholder engagement'/exp OR 'treatment outcome'/exp OR 'total quality management'/exp OR 'program evaluation'/exp OR 'implementation science'/exp OR effective*:ti,ab,kw,de OR efficac*:ti,ab,kw,de OR feasibility:ti,ab,kw,de OR acceptability:ti,ab,kw,de OR implementation:ti,ab,kw,de OR sustainability:ti,ab,kw,de OR adoption:ti,ab,kw,de OR appropriateness:ti,ab,kw,de OR fidelity:ti,ab,kw,de OR "implementation cost*":ti,ab,kw,de OR "patient experience*":ti,ab,kw,de OR "symptom reduction*":ti,ab,kw,de OR applicability:ti,ab,kw,de OR "quality improvement":ti,ab,kw,de OR "program evaluation":ti,ab,kw,de OR "provider perspective*":ti,ab,kw,de OR "provider interview*":ti,ab,kw,de OR "stakeholder perspective*":ti,ab,kw,de OR "stakeholder interview*":ti,ab,kw,de OR "stakeholder feedback":ti,ab,kw,de OR "provider feedback":ti,ab,kw,de OR "end-user input":ti,ab,kw,de OR "symptom change*":ti,ab,kw,de OR "PCL-5":ti,ab,kw,de OR "PTSD checklist":ti,ab,kw,de OR "randomized control trial":ti,ab,kw,de OR "randomized controlled trial*":ti,ab,kw,de OR RCT:ti,ab,kw,de OR "pilot trial":ti,ab,kw,de OR "pilot study":ti,ab,kw,de OR review:ti,ab,kw,de OR "treatment recommendation*":ti,ab,kw,de OR "clinical recommendation*":ti,ab,kw,de OR "treatment consideration*":ti,ab,kw,de OR "treatment recommendation*":ti,ab,kw,de OR "case series":ti,ab,kw,de OR "case stud*":ti,ab,kw,de OR "clinical trial*":ti,ab,kw,de OR "participatory research":ti,ab,kw,de OR "comparative effectiveness":ti,ab,kw,de OR "comparative efficacy":ti,ab,kw,de OR "case conceptualization*":ti,ab,kw,de OR "case formulation*":ti,ab,kw,de OR qualitative:ti,ab,kw,de OR "participatory design":ti,ab,kw,de OR "human-centered design":ti,ab,kw,de OR "human centered design":ti,ab,kw,de OR "community engage*":ti,ab,kw,de OR "focus group*":ti,ab,kw,de OR "focus-group*":ti,ab,kw,de OR "clinical commentar*":ti,ab,kw,de OR editorial*:ti,ab,kw,de OR "clinical consideration*":ti,ab,kw,de) AND [embase]/lim NOT ([embase]/lim AND [medline]/lim) AND [english]/lim *AND [2000-2022]/py*

**PsycINFO Search String**

**PASTEABLE PSYCINFO SEARCH – Use *Advanced Search* (apply search limits date “2000”/language English)**

((exp transsexualism/ OR exp Gender Identity/ OR exp Sexual Minority Groups/ OR exp Transgender/ OR exp Sexual orientation/) OR (bisexual* OR F2M OR ("female-to-male" AND gender ) OR gay OR gays OR "gender dysphori*" OR "gender identit*" OR "gender queer" OR genderqueer OR GLBQ* OR GLBT* OR homosexual* OR intersex OR lesbian* OR lesbigay OR LGB OR LGBT* OR M2F OR MtoF OR FtoM OR ("male-to-female" AND gender) OR queer* OR "sexual and gender minorit*" OR "sexual identit*" OR "sexual minorit*" OR "sexual orientation*" OR "sexual preference*" OR "trans fem*" OR "trans male" OR "transmasc*" OR "trans man" OR "trans men" OR "trans people" OR "trans wom*" OR transgender* OR transexual* OR "bi-umbrella" OR "trans umbrella" OR "trans and gender divers*" OR "gender divers*" OR TGNC OR "non-binary" OR nonbinary OR "gender nonconforming" OR "gender minorit*" OR BGLT* OR pansexual* OR asexual* OR omnisexual* OR "gender fluid*" OR genderfluid* OR "men loving men" OR "women loving women" OR "two spirit" OR aromantic* OR "grey sexual*" OR demisexual* OR demiromantic* OR "sexual minorit*" OR "same sex" OR "same gender" OR agender* OR sapphic OR "gender euphori*" OR panromantic* OR homoromantic* OR biromantic* OR bigender* OR "asexual umbrella" OR "gender divergen*" OR "gender atypical*" OR "gender expression*" OR butch OR femme OR "trans masc*" OR transfem* OR twink* OR "stud community" OR blaQ OR blaQueer OR "gender expansive*" OR "gender variant" OR "masculine of center" OR "feminine of center" OR "multi sexual*" OR "multisexual*" OR neutrois OR omnigender* OR "third gender*" OR polygender* OR "poly gender*" OR pangender* OR "pan gender*" OR polysexual* OR "poly sexual*" OR "romantic orientation*" OR sogie OR "sexual orientation" OR "gender identity" OR "gender expression" OR enby).ti,ab,id,hw)

**AND**

exp 'cognitive behavior therapy'/ OR exp 'psychotherapy'/ OR (Intervention OR "cognitive behavioral therap*" OR "cognitive behavior therap*" OR "cognitive behavioural therap*" OR "cognitive behaviour therap*" OR "TF-CBT" OR "trauma-focused CBT" OR "trauma focused" OR "prolonged exposure therapy" OR "written exposure" OR "cognitive processing therapy" OR CPT OR "exposure therap*" OR psychotherap* OR "talk therap*" OR "cognitive restructur*" OR "cognitive reappraisal" OR "exposure based" OR "trauma informed" OR "evidence based" OR STAIR OR "STAIR narrative therapy" OR "narrative exposure" OR "narrative therapy" OR "peer support*" OR "therapist led" OR "therapist support*" OR "skills coach" OR "dialectical behavioral therapy" OR "dialectical behavior therapy" OR "dialectical behavioural therapy" OR "dialectical behaviour therapy" OR "cognitive therap*" OR "brief eclectic psychotherapy" OR "empirically supported" OR "trauma treatment" OR "relaxation training" OR "skills training and affective interpersonal regulation" OR EMDR OR "eye movement desensitization reprocessing" OR "eye movement desensitization and reprocessing" OR "processing therap*" OR "affirming care" OR "empowerment based" OR "LGBT affirm*" OR "LGBTQ affirm*" OR "trans affirm*" OR "LGBT-affirmative treatment" OR "behavior therap*" OR "behaviour therap*" OR "behavioral therap*" OR "behavioural therap*" OR mindfulness OR "seeking safety" OR "collaborative care" OR "stress inoculation training" OR "present centered therapy" OR "interpersonal psychotherap*" OR "acceptance and commitment therapy" OR "supportive counseling" OR "supportive therap*" OR "supportive treatment" OR "brief intervention*" OR "scalable intervention*" OR "integrated care" OR "cognitive behavioral treatment" OR "cognitive behavior treatment" OR "cognitive behavioural treatment" OR "cognitive behaviour treatment" OR "prolonged exposure treatment" OR "dialectical behavioral treatment" OR "dialectical behaviour treatment" OR "acceptance and commitment therapy" OR "acceptance and commitment treatment" OR "behavior treatment" OR "behavioral treatment" OR "behavioural treatment" OR "behaviour treatment" OR "brief eclectic treatment" OR "flooding" OR "implosive" OR "present-centered therapy" OR "cognitive restructuring" OR "trauma-related guilt reduction" OR "adapted disclosure" OR "empowering queer identities in psychotherapy" OR "effective skills to empower effective men" OR "virtual reality" OR "DBT-PE" OR "interpersonal psychotherapy" OR "accelerated resolution therapy" OR "accelerated resolution treatment" OR "imagery rehearsal therapy" OR "imagery rehearsal treatment" OR "trauma management therapy" OR "trauma management treatment" OR "processing treatment" OR "trauma therapy").ti,ab,id,hw

**AND**

(exp 'microaggression'/ OR exp 'prejudice'/ OR exp 'minority stress'/ OR (prejudice OR "minority stress" OR microaggress* OR concealment OR "concealable identit*" OR discrimination OR homophobia OR biphobia OR transphobia OR harassment OR victimization OR heteronorm* OR homonorm* OR "hate crim*" OR prejudice* OR systemic* OR barriers OR "expectations of rejection" OR "expectation of rejection" OR tokenism OR tokeniz* OR minoritiz* OR marginaliz* OR bias OR burden OR "micro-aggress*" OR macroaggress* OR "macro-aggress*" OR rejection OR lesbophobia OR heterosexis* OR cissexis* OR stigma* OR "traumatic invalidation" OR "family rejection" OR oppression OR oppressive OR outness OR stereotyp*).ti,ab,id,hw)

**OR**

exp posttraumatic stress disorder/ OR (PTSD OR posttrauma* OR trauma* OR "Criterion A stressor*" OR PTS OR avoidance OR "traumatic intrusion*" OR "intrusive thought*" OR "intrusive cognition*" OR "intrusive memor*" OR hyperarousal OR hypervigilenc* OR "post-trauma*" OR "traumatic injury" OR "acute stress disorder*" OR "moral injur*" OR reexperiencing OR "re-experiencing" OR "negative alterations in cognit*" OR "negative alteration in mood" OR "fear structure*").ti,ab,id,hw

**AND**

exp stakeholder/ OR exp 'treatment outcome'/ OR exp 'quality of care'/ OR exp 'program evaluation'/ OR ("treatment outcome*" OR "program evaluation" OR effective* OR efficac* OR feasibility OR acceptability OR implementation OR sustainability OR adoption OR appropriateness OR fidelity OR "implementation cost*" OR "patient experience*" OR "symptom reduction*" OR applicability OR "quality improvement" OR "program evaluation" OR "provider perspective*" OR "provider interview*" OR "stakeholder perspective*" OR "stakeholder interview*" OR "stakeholder feedback" OR "provider feedback" OR "end-user input" OR "symptom change*" OR "PCL-5" OR "PTSD checklist" OR "randomized control trial" OR "randomized controlled trial*" OR RCT OR "pilot trial" OR "pilot study" OR review OR "treatment recommendation*" OR "clinical recommendation*" OR "treatment consideration*" OR "treatment recommendation*" OR "case series" OR "case stud*" OR "clinical trial*" OR "participatory research" OR "comparative effectiveness" OR "comparative efficacy" OR "case conceptualization*" OR "case formulation*" OR qualitative OR "participatory design" OR "human-centered design" OR "human centered design" OR "community engage*" OR "focus group*" OR "focus-group*" OR "clinical commentar*" OR editorial* OR "clinical consideration*").ti,ab,id,hw

**PubMed Search String**

Search: 1 AND 2 AND (3 OR 4) AND 5 AND 6

**1: Population terms**

(transsexualism[MeSH] OR “Sexual and Gender Minorities”[mesh] OR “bisexuality”[mesh] OR “homosexuality”[mesh] OR “transgender persons”[mesh] OR bisexual*[tw] OR F2M[tw] OR (“female-to-male”[tw] AND gender) OR gay[tw] OR gays[tw] OR “gender dysphori*”[tw] OR “gender identit*”[tw] OR “gender queer”[tw] OR genderqueer[tw] OR GLBQ*[tw] OR GLBT*[tw] OR homosexual*[tw] OR intersex[tw] OR lesbian*[tw] OR lesbigay[tw] OR LGB[tw] OR LGBT*[tw] OR M2F[tw] OR MtoF[tw] OR FtoM[tw] OR (“male-to-female”[tw] AND gender) OR queer*[tw] OR “sexual and gender minorit*”[tw] OR “sexual identit*”[tw] OR “sexual minorit*”[tw] OR “sexual orientation*”[tw] OR “sexual preference*”[tw] OR “trans fem*”[tw] OR “trans male”[tw] OR “transmasc*” OR “trans man”[tw] OR “trans men”[tw] OR “trans people”[tw] OR “trans wom*”[tw] OR transgender*[tw] OR transexual*[tw] OR “bi umbrella”[tw] OR “trans umbrella”[tw] OR “trans and gender divers*”[tw] OR “gender divers*”[tw] OR TGNC[tw] OR “non-binary”[tw] OR nonbinary[tw] OR “gender nonconforming”[tw] OR “gender minorit*”[tw] OR BGLT*[tw] OR pansexual*[tw] OR asexual*[tw] OR omnisexual*[tw] OR “gender fluid*”[tw] OR genderfluid*[tw] OR “men loving men”[tw] OR “women loving women”[tw] OR “two spirit”[tw] OR aromantic*[tw] OR “grey sexual*”[tw] OR demisexual*[tw] OR demiromantic*[tw] OR “sexual minorit*”[tw] OR “same sex”[tw] OR “same gender”[tw] OR agender*[tw] OR sapphic[tw] OR “gender euphori*”[tw] OR panromantic*[tw] OR homoromantic*[tw] OR biromantic*[tw] OR bigender*[tw] OR “asexual umbrella”[tw] OR “gender divergen*”[tw] OR “gender atypical*”[tw] OR “gender expression*”[tw] OR butch[tw] OR femme[tw] OR “trans masc*”[tw] OR transfem*[tw] OR twink*[tw] OR “stud community”[tw] OR blaQ[tw] OR blaQueer[tw] OR “gender expansive*”[tw] OR “gender variant”[tw] OR “masculine of center”[tw] “feminine of center”[tw] OR “multi sexual*”[tw] “multisexual*”[tw] OR neutrois[tw] OR omnigender*[tw] OR “third gender*”[tw] OR polygender*[tw] “poly gender*”[tw] OR pangender*[tw] OR “pan gender*”[tw] OR polysexual*[tw] OR “poly sexual*”[tw] OR “romantic orientation*”[tw] OR sogie[tw] OR “sexual orientation” [tw] OR “gender identity” [tw] OR “gender expression”[tw] OR enby [tw])

**2: Treatment Terms**

(“therapy” [sh] OR “psychotherapy”[Mesh] OR "Cognitive Behavioral Therapy"[Mesh] OR Intervention[tw] OR “cognitive behavioral therap*”[tw] OR “cognitive behavior therap*”[tw] OR “cognitive behavioural therap*”[tw] OR “cognitive behaviour therap*”[tw] OR “TF-CBT”[tw] OR “trauma-focused CBT”[tw] OR “trauma focused”[tw] OR “prolonged exposure therapy”[tw] OR “written exposure”[tw] OR “cognitive processing therapy”[tw] OR CPT [tw] OR “exposure therap*”[tw] OR psychotherap*[tw] OR “talk therap*”[tw] OR “cognitive restructur*”[tw] OR “cognitive reappraisal”[tw] OR “exposure based”[tw] OR “trauma informed”[tw] OR “evidence based”[tw] OR STAIR[tw] OR “STAIR narrative therapy”[tw] OR “narrative exposure”[tw] OR “narrative therapy”[tw] OR “peer support*”[tw] OR “therapist led”[tw] OR “therapist support*”[tw] OR “skills coach”[tw] OR “dialectical behavioral therapy”[tw] OR “dialectical behavior therapy”[tw] OR “dialectical behavioural therapy”[tw] OR “dialectical behaviour therapy”[tw] OR “cognitive therap*”[tw] OR “brief eclectic psychotherapy”[tw] OR “empirically supported”[tw] OR “trauma treatment”[tw] OR “relaxation training”[tw] OR “skills training and affective interpersonal regulation”[tw] OR EMDR[tw] OR “eye movement desensitization reprocessing”[tw] OR “eye movement desensitization and reprocessing”[tw] OR “processing therap*”[tw] OR “affirming care”[tw] OR “empowerment based”[tw] OR “LGBT affirm*”[tw] OR “LGBTQ affirm*”[tw] OR “trans affirm*”[tw] OR “LGBT-affirmative treatment”[tw] OR “behavior therap*”[tw] OR “behaviour therap*”[tw] OR “behavioral therap*”[tw] OR “behavioural therap*”[tw] OR mindfulness[tw] OR “seeking safety”[tw] OR “collaborative care”[tw] OR “stress inoculation training”[tw] OR “present centered therapy”[tw] OR “interpersonal psychotherap*”[tw] OR “acceptance and commitment therapy”[tw] OR “supportive counseling”[tw] OR “supportive therap*”[tw] OR “supportive treatment”[tw] OR “brief intervention*”[tw] OR “scalable intervention*”[tw] OR “integrated care”[tw] OR “cognitive behavioral treatment”[tw] OR “cognitive behavior treatment”[tw] OR “cognitive behavioural treatment”[tw] OR “cognitive behaviour treatment” [tw] OR “prolonged exposure treatment” [tw] OR “dialectical behavioral treatment”[tw] OR “dialectical behaviour treatment”[tw] OR “acceptance and commitment therapy”[tw] OR “acceptance and commitment treatment”[tw] OR “behavior treatment”[tw] OR “behavioral treatment”[tw] OR “behavioural treatment”[tw] OR “behaviour treatment”[tw] OR “brief eclectic treatment”[tw] OR “flooding”[tw] OR “implosive”[tw] OR “present-centered therapy”[tw] OR “cognitive restructuring”[tw] OR “trauma-related guilt reduction”[tw] OR “adapted disclosure”[tw] OR “empowering queer identities in psychotherapy”[tw] OR “effective skills to empower effective men”[tw] OR “virtual reality”[tw] OR “DBT-PE”[tw] OR “interpersonal psychotherapy”[tw] OR “accelerated resolution therapy”[tw] OR “accelerated resolution treatment”[tw] OR “imagery rehearsal therapy”[tw] OR “imagery rehearsal treatment”[tw] OR “trauma management therapy”[tw] OR “trauma management treatment”[tw] OR “processing treatment”[tw] OR “trauma therapy”[tw])

**3: Minority Stress Terms**

(“microaggression”[Mesh] OR "Prejudice"[Mesh] OR “minority stress”[tw] OR microaggress*[tw] OR concealment[tw] OR “concealable identit*”[tw] OR discrimination[tw] OR homophobia[tw] OR biphobia[tw] OR transphobia[tw] OR harassment[tw] OR victimization[tw] OR heteronorm*[tw] OR homonorm*[tw] OR “hate crim*”[tw] OR prejudice*[tw] OR systemic*[tw] OR barriers[tw] OR “expectations of rejection”[tw] OR “expectation of rejection”[tw] OR tokenism[tw] tokeniz*[tw] OR minoritiz*[tw] OR marginaliz*[tw] OR bias[tw] OR burden[tw] OR “micro-aggress*”[tw] OR macroaggress*[tw] OR “macro-aggress*”[tw] OR rejection[tw] OR lesbophobia[tw] OR heterosexis*[tw] OR cissexis*[tw] OR stigma*[tw] OR “traumatic invalidation”[tw] OR “family rejection”[tw] OR oppression[tw] OR oppressive[tw] OR outness[tw] OR stereotyp*[tw])

**4: Trauma Terms**

("Trauma and Stressor Related Disorders"[Mesh] OR "Stress Disorders, Post-Traumatic"[Mesh] OR PTSD[tw] OR posttrauma*[tw] OR trauma*[tw] OR “Criterion A stressor*”[tw] OR PTS[tw] OR avoidance[tw] OR “traumatic intrusion*”[tw] OR “intrusive thought*”[tw] OR “intrusive cognition*”[tw] OR “intrusive memor*”[tw] OR hyperarousal[tw] OR hypervigilenc*[tw] OR “post-trauma*”[tw] OR “traumatic injury”[tw] OR “acute stress disorder*”[tw] OR “moral injur*”[tw] OR reexperiencing[tw] OR “re-experiencing”[tw] OR “negative alterations in cognit*” [tw] OR “negative alteration in mood”[tw] OR “fear structure*”[tw])

**5. Outcome & Design Terms**

("Stakeholder Participation"[Mesh] OR "Treatment Outcome"[Mesh] OR "Program Evaluation"[Mesh] OR "Quality Improvement"[Mesh] OR "Implementation Science"[Mesh] OR “treatment outcome*” [mesh] OR effective*[tw] OR efficac*[tw] OR feasibility[tw] OR acceptability[tw] OR implementation[tw] OR sustainability[tw] OR adoption[tw] OR appropriateness[tw] OR fidelity[tw] OR “implementation cost*”[tw] OR “patient experience*”[tw] OR “symptom reduction*”[tw] OR applicability[tw] OR “quality improvement”[tw] OR “program evaluation”[tw] OR “provider perspective*”[tw] OR “provider interview*”[tw] OR “stakeholder perspective*”[tw] OR “stakeholder interview*”[tw] OR “stakeholder feedback”[tw] OR “provider feedback”[tw] OR “end-user input”[tw] OR “symptom change*”[tw] OR “PCL-5”[tw] OR “PTSD checklist”[tw] OR “randomized control trial”[tw] OR “randomized controlled trial*”[tw] OR RCT[tw] OR “pilot trial”[tw] OR “pilot study”[tw] OR review[tw] OR “treatment recommendation*”[tw] OR “clinical recommendation*”[tw] OR “treatment consideration*”[tw] OR “treatment recommendation*”[tw] OR “case series”[tw] OR “case stud*”[tw] OR “clinical trial*”[tw] OR “participatory research”[tw] OR “comparative effectiveness”[tw] OR “comparative efficacy”[tw] OR “case conceptualization*”[tw] OR “case formulation*”[tw] OR qualitative[tw] OR “participatory design”[tw] OR “human-centered design”[tw] OR “human centered design”[tw] OR “community engage*”[tw] OR “focus group*”[tw] OR “focus-group*”[tw] OR “clinical commentar*”[tw] OR editorial*[tw] OR “clinical consideration*”[tw])

**6. Filters**

AND english [lang] AND 2000:3000 [dp]

--

SEARCH #1: *1 AND 2 AND 3 AND 5 AND 6*

((transsexualism[MeSH] OR "Sexual and Gender Minorities"[mesh] OR "bisexuality"[mesh] OR "homosexuality"[mesh] OR "transgender persons"[mesh] OR bisexual*[tw] OR F2M[tw] OR ("female-to-male"[tw] AND gender) OR gay[tw] OR gays[tw] OR "gender dysphori*"[tw] OR "gender identit*"[tw] OR "gender queer"[tw] OR genderqueer[tw] OR GLBQ*[tw] OR GLBT*[tw] OR homosexual*[tw] OR intersex[tw] OR lesbian*[tw] OR lesbigay[tw] OR LGB[tw] OR LGBT*[tw] OR M2F[tw] OR MtoF[tw] OR FtoM[tw] OR ("male-to-female"[tw] AND gender) OR queer*[tw] OR "sexual and gender minorit*"[tw] OR "sexual identit*"[tw] OR "sexual minorit*"[tw] OR "sexual orientation*"[tw] OR "sexual preference*"[tw] OR "trans fem*"[tw] OR "trans male"[tw] OR "transmasc*" OR "trans man"[tw] OR "trans men"[tw] OR "trans people"[tw] OR "trans wom*"[tw] OR transgender*[tw] OR transexual*[tw] OR "bi umbrella"[tw] OR "trans umbrella"[tw] OR "trans and gender divers*"[tw] OR "gender divers*"[tw] OR TGNC[tw] OR "non-binary"[tw] OR nonbinary[tw] OR "gender nonconforming"[tw] OR "gender minorit*"[tw] OR BGLT*[tw] OR pansexual*[tw] OR asexual*[tw] OR omnisexual*[tw] OR "gender fluid*"[tw] OR genderfluid*[tw] OR "men loving men"[tw] OR "women loving women"[tw] OR "two spirit"[tw] OR aromantic*[tw] OR "grey sexual*"[tw] OR demisexual*[tw] OR demiromantic*[tw] OR "sexual minorit*"[tw] OR "same sex"[tw] OR "same gender"[tw] OR agender*[tw] OR sapphic[tw] OR "gender euphori*"[tw] OR panromantic*[tw] OR homoromantic*[tw] OR biromantic*[tw] OR bigender*[tw] OR "asexual umbrella"[tw] OR "gender divergen*"[tw] OR "gender atypical*"[tw] OR "gender expression*"[tw] OR butch[tw] OR femme[tw] OR "trans masc*"[tw] OR transfem*[tw] OR twink*[tw] OR "stud community"[tw] OR blaQ[tw] OR blaQueer[tw] OR "gender expansive*"[tw] OR "gender variant"[tw] OR "masculine of center"[tw] "feminine of center"[tw] OR "multi sexual*"[tw] "multisexual*"[tw] OR neutrois[tw] OR omnigender*[tw] OR "third gender*"[tw] OR polygender*[tw] "poly gender*"[tw] OR pangender*[tw] OR "pan gender*"[tw] OR polysexual*[tw] OR "poly sexual*"[tw] OR "romantic orientation*"[tw] OR sogie[tw] OR "sexual orientation" [tw] OR "gender identity" [tw] OR "gender expression"[tw] OR enby [tw])) AND (("therapy" [sh] OR "psychotherapy"[Mesh] OR "Cognitive Behavioral Therapy"[Mesh] OR Intervention[tw] OR "cognitive behavioral therap*"[tw] OR "cognitive behavior therap*"[tw] OR "cognitive behavioural therap*"[tw] OR "cognitive behaviour therap*"[tw] OR "TF-CBT"[tw] OR "trauma-focused CBT"[tw] OR "trauma focused"[tw] OR "prolonged exposure therapy"[tw] OR "written exposure"[tw] OR "cognitive processing therapy"[tw] OR CPT [tw] OR "exposure therap*"[tw] OR psychotherap*[tw] OR "talk therap*"[tw] OR "cognitive restructur*"[tw] OR "cognitive reappraisal"[tw] OR "exposure based"[tw] OR "trauma informed"[tw] OR "evidence based"[tw] OR STAIR[tw] OR "STAIR narrative therapy"[tw] OR "narrative exposure"[tw] OR "narrative therapy"[tw] OR "peer support*"[tw] OR "therapist led"[tw] OR "therapist support*"[tw] OR "skills coach"[tw] OR "dialectical behavioral therapy"[tw] OR "dialectical behavior therapy"[tw] OR "dialectical behavioural therapy"[tw] OR "dialectical behaviour therapy"[tw] OR "cognitive therap*"[tw] OR "brief eclectic psychotherapy"[tw] OR "empirically supported"[tw] OR "trauma treatment"[tw] OR "relaxation training"[tw] OR "skills training and affective interpersonal regulation"[tw] OR EMDR[tw] OR "eye movement desensitization reprocessing"[tw] OR "eye movement desensitization and reprocessing"[tw] OR "processing therap*"[tw] OR "affirming care"[tw] OR "empowerment based"[tw] OR "LGBT affirm*"[tw] OR "LGBTQ affirm*"[tw] OR "trans affirm*"[tw] OR "LGBT-affirmative treatment"[tw] OR "behavior therap*"[tw] OR "behaviour therap*"[tw] OR "behavioral therap*"[tw] OR "behavioural therap*"[tw] OR mindfulness[tw] OR "seeking safety"[tw] OR "collaborative care"[tw] OR "stress inoculation training"[tw] OR "present centered therapy"[tw] OR "interpersonal psychotherap*"[tw] OR "acceptance and commitment therapy"[tw] OR "supportive counseling"[tw] OR "supportive therap*"[tw] OR "supportive treatment"[tw] OR "brief intervention*"[tw] OR "scalable intervention*"[tw] OR "integrated care"[tw] OR "cognitive behavioral treatment"[tw] OR "cognitive behavior treatment"[tw] OR "cognitive behavioural treatment"[tw] OR "cognitive behaviour treatment" [tw] OR "prolonged exposure treatment" [tw] OR "dialectical behavioral treatment"[tw] OR "dialectical behaviour treatment"[tw] OR "acceptance and commitment therapy"[tw] OR "acceptance and commitment treatment"[tw] OR "behavior treatment"[tw] OR "behavioral treatment"[tw] OR "behavioural treatment"[tw] OR "behaviour treatment"[tw] OR "brief eclectic treatment"[tw] OR "flooding"[tw] OR "implosive"[tw] OR "present-centered therapy"[tw] OR "cognitive restructuring"[tw] OR "trauma-related guilt reduction"[tw] OR "adapted disclosure"[tw] OR "empowering queer identities in psychotherapy"[tw] OR "effective skills to empower effective men"[tw] OR "virtual reality"[tw] OR "DBT-PE"[tw] OR "interpersonal psychotherapy"[tw] OR "accelerated resolution therapy"[tw] OR "accelerated resolution treatment"[tw] OR "imagery rehearsal therapy"[tw] OR "imagery rehearsal treatment"[tw] OR "trauma management therapy"[tw] OR "trauma management treatment"[tw] OR "processing treatment"[tw] OR "trauma therapy"[tw])) AND (("microaggression"[Mesh] OR "Prejudice"[Mesh] OR "minority stress"[tw] OR microaggress*[tw] OR concealment[tw] OR "concealable identit*"[tw] OR discrimination[tw] OR homophobia[tw] OR biphobia[tw] OR transphobia[tw] OR harassment[tw] OR victimization[tw] OR heteronorm*[tw] OR homonorm*[tw] OR "hate crim*"[tw] OR prejudice*[tw] OR systemic*[tw] OR barriers[tw] OR "expectations of rejection"[tw] OR "expectation of rejection"[tw] OR tokenism[tw] tokeniz*[tw] OR minoritiz*[tw] OR marginaliz*[tw] OR bias[tw] OR burden[tw] OR "micro-aggress*"[tw] OR macroaggress*[tw] OR "macro-aggress*"[tw] OR rejection[tw] OR lesbophobia[tw] OR heterosexis*[tw] OR cissexis*[tw] OR stigma*[tw] OR "traumatic invalidation"[tw] OR "family rejection"[tw] OR oppression[tw] OR oppressive[tw] OR outness[tw] OR stereotyp*[tw])) AND (("Stakeholder Participation"[Mesh] OR "Treatment Outcome"[Mesh] OR "Program Evaluation"[Mesh] OR "Quality Improvement"[Mesh] OR "Implementation Science"[Mesh] OR "treatment outcome*" [mesh] OR effective*[tw] OR efficac*[tw] OR feasibility[tw] OR acceptability[tw] OR implementation[tw] OR sustainability[tw] OR adoption[tw] OR appropriateness[tw] OR fidelity[tw] OR "implementation cost*"[tw] OR "patient experience*"[tw] OR "symptom reduction*"[tw] OR applicability[tw] OR "quality improvement"[tw] OR "program evaluation"[tw] OR "provider perspective*"[tw] OR "provider interview*"[tw] OR "stakeholder perspective*"[tw] OR "stakeholder interview*"[tw] OR "stakeholder feedback"[tw] OR "provider feedback"[tw] OR "end-user input"[tw] OR "symptom change*"[tw] OR "PCL-5"[tw] OR "PTSD checklist"[tw] OR "randomized control trial"[tw] OR "randomized controlled trial*"[tw] OR RCT[tw] OR "pilot trial"[tw] OR "pilot study"[tw] OR review[tw] OR "treatment recommendation*"[tw] OR "clinical recommendation*"[tw] OR "treatment consideration*"[tw] OR "treatment recommendation*"[tw] OR "case series"[tw] OR "case stud*"[tw] OR "clinical trial*"[tw] OR "participatory research"[tw] OR "comparative effectiveness"[tw] OR "comparative efficacy"[tw] OR "case conceptualization*"[tw] OR "case formulation*"[tw] OR qualitative[tw] OR "participatory design"[tw] OR "human-centered design"[tw] OR "human centered design"[tw] OR "community engage*"[tw] OR "focus group*"[tw] OR "focus-group*"[tw] OR "clinical commentar*"[tw] OR editorial*[tw] OR "clinical consideration*"[tw])) AND english [lang] AND 2000:3000 [dp]

SEARCH #2: *1 AND 2 AND 4 AND 5 AND 6*

((transsexualism[MeSH] OR "Sexual and Gender Minorities"[mesh] OR "bisexuality"[mesh] OR "homosexuality"[mesh] OR "transgender persons"[mesh] OR bisexual*[tw] OR F2M[tw] OR ("female-to-male"[tw] AND gender) OR gay[tw] OR gays[tw] OR "gender dysphori*"[tw] OR "gender identit*"[tw] OR "gender queer"[tw] OR genderqueer[tw] OR GLBQ*[tw] OR GLBT*[tw] OR homosexual*[tw] OR intersex[tw] OR lesbian*[tw] OR lesbigay[tw] OR LGB[tw] OR LGBT*[tw] OR M2F[tw] OR MtoF[tw] OR FtoM[tw] OR ("male-to-female"[tw] AND gender) OR queer*[tw] OR "sexual and gender minorit*"[tw] OR "sexual identit*"[tw] OR "sexual minorit*"[tw] OR "sexual orientation*"[tw] OR "sexual preference*"[tw] OR "trans fem*"[tw] OR "trans male"[tw] OR "transmasc*" OR "trans man"[tw] OR "trans men"[tw] OR "trans people"[tw] OR "trans wom*"[tw] OR transgender*[tw] OR transexual*[tw] OR "bi umbrella"[tw] OR "trans umbrella"[tw] OR "trans and gender divers*"[tw] OR "gender divers*"[tw] OR TGNC[tw] OR "non-binary"[tw] OR nonbinary[tw] OR "gender nonconforming"[tw] OR "gender minorit*"[tw] OR BGLT*[tw] OR pansexual*[tw] OR asexual*[tw] OR omnisexual*[tw] OR "gender fluid*"[tw] OR genderfluid*[tw] OR "men loving men"[tw] OR "women loving women"[tw] OR "two spirit"[tw] OR aromantic*[tw] OR "grey sexual*"[tw] OR demisexual*[tw] OR demiromantic*[tw] OR "sexual minorit*"[tw] OR "same sex"[tw] OR "same gender"[tw] OR agender*[tw] OR sapphic[tw] OR "gender euphori*"[tw] OR panromantic*[tw] OR homoromantic*[tw] OR biromantic*[tw] OR bigender*[tw] OR "asexual umbrella"[tw] OR "gender divergen*"[tw] OR "gender atypical*"[tw] OR "gender expression*"[tw] OR butch[tw] OR femme[tw] OR "trans masc*"[tw] OR transfem*[tw] OR twink*[tw] OR "stud community"[tw] OR blaQ[tw] OR blaQueer[tw] OR "gender expansive*"[tw] OR "gender variant"[tw] OR "masculine of center"[tw] "feminine of center"[tw] OR "multi sexual*"[tw] "multisexual*"[tw] OR neutrois[tw] OR omnigender*[tw] OR "third gender*"[tw] OR polygender*[tw] "poly gender*"[tw] OR pangender*[tw] OR "pan gender*"[tw] OR polysexual*[tw] OR "poly sexual*"[tw] OR "romantic orientation*"[tw] OR sogie[tw] OR "sexual orientation" [tw] OR "gender identity" [tw] OR "gender expression"[tw] OR enby [tw])) AND (("therapy" [sh] OR "psychotherapy"[Mesh] OR "Cognitive Behavioral Therapy"[Mesh] OR Intervention[tw] OR "cognitive behavioral therap*"[tw] OR "cognitive behavior therap*"[tw] OR "cognitive behavioural therap*"[tw] OR "cognitive behaviour therap*"[tw] OR "TF-CBT"[tw] OR "trauma-focused CBT"[tw] OR "trauma focused"[tw] OR "prolonged exposure therapy"[tw] OR "written exposure"[tw] OR "cognitive processing therapy"[tw] OR CPT [tw] OR "exposure therap*"[tw] OR psychotherap*[tw] OR "talk therap*"[tw] OR "cognitive restructur*"[tw] OR "cognitive reappraisal"[tw] OR "exposure based"[tw] OR "trauma informed"[tw] OR "evidence based"[tw] OR STAIR[tw] OR "STAIR narrative therapy"[tw] OR "narrative exposure"[tw] OR "narrative therapy"[tw] OR "peer support*"[tw] OR "therapist led"[tw] OR "therapist support*"[tw] OR "skills coach"[tw] OR "dialectical behavioral therapy"[tw] OR "dialectical behavior therapy"[tw] OR "dialectical behavioural therapy"[tw] OR "dialectical behaviour therapy"[tw] OR "cognitive therap*"[tw] OR "brief eclectic psychotherapy"[tw] OR "empirically supported"[tw] OR "trauma treatment"[tw] OR "relaxation training"[tw] OR "skills training and affective interpersonal regulation"[tw] OR EMDR[tw] OR "eye movement desensitization reprocessing"[tw] OR "eye movement desensitization and reprocessing"[tw] OR "processing therap*"[tw] OR "affirming care"[tw] OR "empowerment based"[tw] OR "LGBT affirm*"[tw] OR "LGBTQ affirm*"[tw] OR "trans affirm*"[tw] OR "LGBT-affirmative treatment"[tw] OR "behavior therap*"[tw] OR "behaviour therap*"[tw] OR "behavioral therap*"[tw] OR "behavioural therap*"[tw] OR mindfulness[tw] OR "seeking safety"[tw] OR "collaborative care"[tw] OR "stress inoculation training"[tw] OR "present centered therapy"[tw] OR "interpersonal psychotherap*"[tw] OR "acceptance and commitment therapy"[tw] OR "supportive counseling"[tw] OR "supportive therap*"[tw] OR "supportive treatment"[tw] OR "brief intervention*"[tw] OR "scalable intervention*"[tw] OR "integrated care"[tw] OR "cognitive behavioral treatment"[tw] OR "cognitive behavior treatment"[tw] OR "cognitive behavioural treatment"[tw] OR "cognitive behaviour treatment" [tw] OR "prolonged exposure treatment" [tw] OR "dialectical behavioral treatment"[tw] OR "dialectical behaviour treatment"[tw] OR "acceptance and commitment therapy"[tw] OR "acceptance and commitment treatment"[tw] OR "behavior treatment"[tw] OR "behavioral treatment"[tw] OR "behavioural treatment"[tw] OR "behaviour treatment"[tw] OR "brief eclectic treatment"[tw] OR "flooding"[tw] OR "implosive"[tw] OR "present-centered therapy"[tw] OR "cognitive restructuring"[tw] OR "trauma-related guilt reduction"[tw] OR "adapted disclosure"[tw] OR "empowering queer identities in psychotherapy"[tw] OR "effective skills to empower effective men"[tw] OR "virtual reality"[tw] OR "DBT-PE"[tw] OR "interpersonal psychotherapy"[tw] OR "accelerated resolution therapy"[tw] OR "accelerated resolution treatment"[tw] OR "imagery rehearsal therapy"[tw] OR "imagery rehearsal treatment"[tw] OR "trauma management therapy"[tw] OR "trauma management treatment"[tw] OR "processing treatment"[tw] OR "trauma therapy"[tw])) AND (("Trauma and Stressor Related Disorders"[Mesh] OR "Stress Disorders, Post-Traumatic"[Mesh] OR PTSD[tw] OR posttrauma*[tw] OR trauma*[tw] OR "Criterion A stressor*"[tw] OR PTS[tw] OR avoidance[tw] OR "traumatic intrusion*"[tw] OR "intrusive thought*"[tw] OR "intrusive cognition*"[tw] OR "intrusive memor*"[tw] OR hyperarousal[tw] OR hypervigilenc*[tw] OR "post-trauma*"[tw] OR "traumatic injury"[tw] OR "acute stress disorder*"[tw] OR "moral injur*"[tw] OR reexperiencing[tw] OR "re-experiencing"[tw] OR "negative alterations in cognit*" [tw] OR "negative alteration in mood"[tw] OR "fear structure*"[tw])) AND (("Stakeholder Participation"[Mesh] OR "Treatment Outcome"[Mesh] OR "Program Evaluation"[Mesh] OR "Quality Improvement"[Mesh] OR "Implementation Science"[Mesh] OR "treatment outcome*" [mesh] OR effective*[tw] OR efficac*[tw] OR feasibility[tw] OR acceptability[tw] OR implementation[tw] OR sustainability[tw] OR adoption[tw] OR appropriateness[tw] OR fidelity[tw] OR "implementation cost*"[tw] OR "patient experience*"[tw] OR "symptom reduction*"[tw] OR applicability[tw] OR "quality improvement"[tw] OR "program evaluation"[tw] OR "provider perspective*"[tw] OR "provider interview*"[tw] OR "stakeholder perspective*"[tw] OR "stakeholder interview*"[tw] OR "stakeholder feedback"[tw] OR "provider feedback"[tw] OR "end-user input"[tw] OR "symptom change*"[tw] OR "PCL-5"[tw] OR "PTSD checklist"[tw] OR "randomized control trial"[tw] OR "randomized controlled trial*"[tw] OR RCT[tw] OR "pilot trial"[tw] OR "pilot study"[tw] OR review[tw] OR "treatment recommendation*"[tw] OR "clinical recommendation*"[tw] OR "treatment consideration*"[tw] OR "treatment recommendation*"[tw] OR "case series"[tw] OR "case stud*"[tw] OR "clinical trial*"[tw] OR "participatory research"[tw] OR "comparative effectiveness"[tw] OR "comparative efficacy"[tw] OR "case conceptualization*"[tw] OR "case formulation*"[tw] OR qualitative[tw] OR "participatory design"[tw] OR "human-centered design"[tw] OR "human centered design"[tw] OR "community engage*"[tw] OR "focus group*"[tw] OR "focus-group*"[tw] OR "clinical commentar*"[tw] OR editorial*[tw] OR "clinical consideration*"[tw])) AND english [lang] AND 2000:3000 [dp]

SEARCH 3: *1 AND 2 AND (3 OR 4) AND 5 AND 6*

(((transsexualism[MeSH] OR "Sexual and Gender Minorities"[mesh] OR "bisexuality"[mesh] OR "homosexuality"[mesh] OR "transgender persons"[mesh] OR bisexual*[tw] OR F2M[tw] OR ("female-to-male"[tw] AND gender) OR gay[tw] OR gays[tw] OR "gender dysphori*"[tw] OR "gender identit*"[tw] OR "gender queer"[tw] OR genderqueer[tw] OR GLBQ*[tw] OR GLBT*[tw] OR homosexual*[tw] OR intersex[tw] OR lesbian*[tw] OR lesbigay[tw] OR LGB[tw] OR LGBT*[tw] OR M2F[tw] OR MtoF[tw] OR FtoM[tw] OR ("male-to-female"[tw] AND gender) OR queer*[tw] OR "sexual and gender minorit*"[tw] OR "sexual identit*"[tw] OR "sexual minorit*"[tw] OR "sexual orientation*"[tw] OR "sexual preference*"[tw] OR "trans fem*"[tw] OR "trans male"[tw] OR "transmasc*" OR "trans man"[tw] OR "trans men"[tw] OR "trans people"[tw] OR "trans wom*"[tw] OR transgender*[tw] OR transexual*[tw] OR "bi umbrella"[tw] OR "trans umbrella"[tw] OR "trans and gender divers*"[tw] OR "gender divers*"[tw] OR TGNC[tw] OR "non-binary"[tw] OR nonbinary[tw] OR "gender nonconforming"[tw] OR "gender minorit*"[tw] OR BGLT*[tw] OR pansexual*[tw] OR asexual*[tw] OR omnisexual*[tw] OR "gender fluid*"[tw] OR genderfluid*[tw] OR "men loving men"[tw] OR "women loving women"[tw] OR "two spirit"[tw] OR aromantic*[tw] OR "grey sexual*"[tw] OR demisexual*[tw] OR demiromantic*[tw] OR "sexual minorit*"[tw] OR "same sex"[tw] OR "same gender"[tw] OR agender*[tw] OR sapphic[tw] OR "gender euphori*"[tw] OR panromantic*[tw] OR homoromantic*[tw] OR biromantic*[tw] OR bigender*[tw] OR "asexual umbrella"[tw] OR "gender divergen*"[tw] OR "gender atypical*"[tw] OR "gender expression*"[tw] OR butch[tw] OR femme[tw] OR "trans masc*"[tw] OR transfem*[tw] OR twink*[tw] OR "stud community"[tw] OR blaQ[tw] OR blaQueer[tw] OR "gender expansive*"[tw] OR "gender variant"[tw] OR "masculine of center"[tw] "feminine of center"[tw] OR "multi sexual*"[tw] "multisexual*"[tw] OR neutrois[tw] OR omnigender*[tw] OR "third gender*"[tw] OR polygender*[tw] "poly gender*"[tw] OR pangender*[tw] OR "pan gender*"[tw] OR polysexual*[tw] OR "poly sexual*"[tw] OR "romantic orientation*"[tw] OR sogie[tw] OR "sexual orientation" [tw] OR "gender identity" [tw] OR "gender expression"[tw] OR enby [tw])) AND (("therapy" [sh] OR "psychotherapy"[Mesh] OR "Cognitive Behavioral Therapy"[Mesh] OR Intervention[tw] OR "cognitive behavioral therap*"[tw] OR "cognitive behavior therap*"[tw] OR "cognitive behavioural therap*"[tw] OR "cognitive behaviour therap*"[tw] OR "TF-CBT"[tw] OR "trauma-focused CBT"[tw] OR "trauma focused"[tw] OR "prolonged exposure therapy"[tw] OR "written exposure"[tw] OR "cognitive processing therapy"[tw] OR CPT [tw] OR "exposure therap*"[tw] OR psychotherap*[tw] OR "talk therap*"[tw] OR "cognitive restructur*"[tw] OR "cognitive reappraisal"[tw] OR "exposure based"[tw] OR "trauma informed"[tw] OR "evidence based"[tw] OR STAIR[tw] OR "STAIR narrative therapy"[tw] OR "narrative exposure"[tw] OR "narrative therapy"[tw] OR "peer support*"[tw] OR "therapist led"[tw] OR "therapist support*"[tw] OR "skills coach"[tw] OR "dialectical behavioral therapy"[tw] OR "dialectical behavior therapy"[tw] OR "dialectical behavioural therapy"[tw] OR "dialectical behaviour therapy"[tw] OR "cognitive therap*"[tw] OR "brief eclectic psychotherapy"[tw] OR "empirically supported"[tw] OR "trauma treatment"[tw] OR "relaxation training"[tw] OR "skills training and affective interpersonal regulation"[tw] OR EMDR[tw] OR "eye movement desensitization reprocessing"[tw] OR "eye movement desensitization and reprocessing"[tw] OR "processing therap*"[tw] OR "affirming care"[tw] OR "empowerment based"[tw] OR "LGBT affirm*"[tw] OR "LGBTQ affirm*"[tw] OR "trans affirm*"[tw] OR "LGBT-affirmative treatment"[tw] OR "behavior therap*"[tw] OR "behaviour therap*"[tw] OR "behavioral therap*"[tw] OR "behavioural therap*"[tw] OR mindfulness[tw] OR "seeking safety"[tw] OR "collaborative care"[tw] OR "stress inoculation training"[tw] OR "present centered therapy"[tw] OR "interpersonal psychotherap*"[tw] OR "acceptance and commitment therapy"[tw] OR "supportive counseling"[tw] OR "supportive therap*"[tw] OR "supportive treatment"[tw] OR "brief intervention*"[tw] OR "scalable intervention*"[tw] OR "integrated care"[tw] OR "cognitive behavioral treatment"[tw] OR "cognitive behavior treatment"[tw] OR "cognitive behavioural treatment"[tw] OR "cognitive behaviour treatment" [tw] OR "prolonged exposure treatment" [tw] OR "dialectical behavioral treatment"[tw] OR "dialectical behaviour treatment"[tw] OR "acceptance and commitment therapy"[tw] OR "acceptance and commitment treatment"[tw] OR "behavior treatment"[tw] OR "behavioral treatment"[tw] OR "behavioural treatment"[tw] OR "behaviour treatment"[tw] OR "brief eclectic treatment"[tw] OR "flooding"[tw] OR "implosive"[tw] OR "present-centered therapy"[tw] OR "cognitive restructuring"[tw] OR "trauma-related guilt reduction"[tw] OR "adapted disclosure"[tw] OR "empowering queer identities in psychotherapy"[tw] OR "effective skills to empower effective men"[tw] OR "virtual reality"[tw] OR "DBT-PE"[tw] OR "interpersonal psychotherapy"[tw] OR "accelerated resolution therapy"[tw] OR "accelerated resolution treatment"[tw] OR "imagery rehearsal therapy"[tw] OR "imagery rehearsal treatment"[tw] OR "trauma management therapy"[tw] OR "trauma management treatment"[tw] OR "processing treatment"[tw] OR "trauma therapy"[tw])) AND (("microaggression"[Mesh] OR "Prejudice"[Mesh] OR "minority stress"[tw] OR microaggress*[tw] OR concealment[tw] OR "concealable identit*"[tw] OR discrimination[tw] OR homophobia[tw] OR biphobia[tw] OR transphobia[tw] OR harassment[tw] OR victimization[tw] OR heteronorm*[tw] OR homonorm*[tw] OR "hate crim*"[tw] OR prejudice*[tw] OR systemic*[tw] OR barriers[tw] OR "expectations of rejection"[tw] OR "expectation of rejection"[tw] OR tokenism[tw] tokeniz*[tw] OR minoritiz*[tw] OR marginaliz*[tw] OR bias[tw] OR burden[tw] OR "micro-aggress*"[tw] OR macroaggress*[tw] OR "macro-aggress*"[tw] OR rejection[tw] OR lesbophobia[tw] OR heterosexis*[tw] OR cissexis*[tw] OR stigma*[tw] OR "traumatic invalidation"[tw] OR "family rejection"[tw] OR oppression[tw] OR oppressive[tw] OR outness[tw] OR stereotyp*[tw])) AND (("Stakeholder Participation"[Mesh] OR "Treatment Outcome"[Mesh] OR "Program Evaluation"[Mesh] OR "Quality Improvement"[Mesh] OR "Implementation Science"[Mesh] OR "treatment outcome*" [mesh] OR effective*[tw] OR efficac*[tw] OR feasibility[tw] OR acceptability[tw] OR implementation[tw] OR sustainability[tw] OR adoption[tw] OR appropriateness[tw] OR fidelity[tw] OR "implementation cost*"[tw] OR "patient experience*"[tw] OR "symptom reduction*"[tw] OR applicability[tw] OR "quality improvement"[tw] OR "program evaluation"[tw] OR "provider perspective*"[tw] OR "provider interview*"[tw] OR "stakeholder perspective*"[tw] OR "stakeholder interview*"[tw] OR "stakeholder feedback"[tw] OR "provider feedback"[tw] OR "end-user input"[tw] OR "symptom change*"[tw] OR "PCL-5"[tw] OR "PTSD checklist"[tw] OR "randomized control trial"[tw] OR "randomized controlled trial*"[tw] OR RCT[tw] OR "pilot trial"[tw] OR "pilot study"[tw] OR review[tw] OR "treatment recommendation*"[tw] OR "clinical recommendation*"[tw] OR "treatment consideration*"[tw] OR "treatment recommendation*"[tw] OR "case series"[tw] OR "case stud*"[tw] OR "clinical trial*"[tw] OR "participatory research"[tw] OR "comparative effectiveness"[tw] OR "comparative efficacy"[tw] OR "case conceptualization*"[tw] OR "case formulation*"[tw] OR qualitative[tw] OR "participatory design"[tw] OR "human-centered design"[tw] OR "human centered design"[tw] OR "community engage*"[tw] OR "focus group*"[tw] OR "focus-group*"[tw] OR "clinical commentar*"[tw] OR editorial*[tw] OR "clinical consideration*"[tw])) AND english [lang] AND 2000:3000 [dp]) OR (((transsexualism[MeSH] OR "Sexual and Gender Minorities"[mesh] OR "bisexuality"[mesh] OR "homosexuality"[mesh] OR "transgender persons"[mesh] OR bisexual*[tw] OR F2M[tw] OR ("female-to-male"[tw] AND gender) OR gay[tw] OR gays[tw] OR "gender dysphori*"[tw] OR "gender identit*"[tw] OR "gender queer"[tw] OR genderqueer[tw] OR GLBQ*[tw] OR GLBT*[tw] OR homosexual*[tw] OR intersex[tw] OR lesbian*[tw] OR lesbigay[tw] OR LGB[tw] OR LGBT*[tw] OR M2F[tw] OR MtoF[tw] OR FtoM[tw] OR ("male-to-female"[tw] AND gender) OR queer*[tw] OR "sexual and gender minorit*"[tw] OR "sexual identit*"[tw] OR "sexual minorit*"[tw] OR "sexual orientation*"[tw] OR "sexual preference*"[tw] OR "trans fem*"[tw] OR "trans male"[tw] OR "transmasc*" OR "trans man"[tw] OR "trans men"[tw] OR "trans people"[tw] OR "trans wom*"[tw] OR transgender*[tw] OR transexual*[tw] OR "bi umbrella"[tw] OR "trans umbrella"[tw] OR "trans and gender divers*"[tw] OR "gender divers*"[tw] OR TGNC[tw] OR "non-binary"[tw] OR nonbinary[tw] OR "gender nonconforming"[tw] OR "gender minorit*"[tw] OR BGLT*[tw] OR pansexual*[tw] OR asexual*[tw] OR omnisexual*[tw] OR "gender fluid*"[tw] OR genderfluid*[tw] OR "men loving men"[tw] OR "women loving women"[tw] OR "two spirit"[tw] OR aromantic*[tw] OR "grey sexual*"[tw] OR demisexual*[tw] OR demiromantic*[tw] OR "sexual minorit*"[tw] OR "same sex"[tw] OR "same gender"[tw] OR agender*[tw] OR sapphic[tw] OR "gender euphori*"[tw] OR panromantic*[tw] OR homoromantic*[tw] OR biromantic*[tw] OR bigender*[tw] OR "asexual umbrella"[tw] OR "gender divergen*"[tw] OR "gender atypical*"[tw] OR "gender expression*"[tw] OR butch[tw] OR femme[tw] OR "trans masc*"[tw] OR transfem*[tw] OR twink*[tw] OR "stud community"[tw] OR blaQ[tw] OR blaQueer[tw] OR "gender expansive*"[tw] OR "gender variant"[tw] OR "masculine of center"[tw] "feminine of center"[tw] OR "multi sexual*"[tw] "multisexual*"[tw] OR neutrois[tw] OR omnigender*[tw] OR "third gender*"[tw] OR polygender*[tw] "poly gender*"[tw] OR pangender*[tw] OR "pan gender*"[tw] OR polysexual*[tw] OR "poly sexual*"[tw] OR "romantic orientation*"[tw] OR sogie[tw] OR "sexual orientation" [tw] OR "gender identity" [tw] OR "gender expression"[tw] OR enby [tw])) AND (("therapy" [sh] OR "psychotherapy"[Mesh] OR "Cognitive Behavioral Therapy"[Mesh] OR Intervention[tw] OR "cognitive behavioral therap*"[tw] OR "cognitive behavior therap*"[tw] OR "cognitive behavioural therap*"[tw] OR "cognitive behaviour therap*"[tw] OR "TF-CBT"[tw] OR "trauma-focused CBT"[tw] OR "trauma focused"[tw] OR "prolonged exposure therapy"[tw] OR "written exposure"[tw] OR "cognitive processing therapy"[tw] OR CPT [tw] OR "exposure therap*"[tw] OR psychotherap*[tw] OR "talk therap*"[tw] OR "cognitive restructur*"[tw] OR "cognitive reappraisal"[tw] OR "exposure based"[tw] OR "trauma informed"[tw] OR "evidence based"[tw] OR STAIR[tw] OR "STAIR narrative therapy"[tw] OR "narrative exposure"[tw] OR "narrative therapy"[tw] OR "peer support*"[tw] OR "therapist led"[tw] OR "therapist support*"[tw] OR "skills coach"[tw] OR "dialectical behavioral therapy"[tw] OR "dialectical behavior therapy"[tw] OR "dialectical behavioural therapy"[tw] OR "dialectical behaviour therapy"[tw] OR "cognitive therap*"[tw] OR "brief eclectic psychotherapy"[tw] OR "empirically supported"[tw] OR "trauma treatment"[tw] OR "relaxation training"[tw] OR "skills training and affective interpersonal regulation"[tw] OR EMDR[tw] OR "eye movement desensitization reprocessing"[tw] OR "eye movement desensitization and reprocessing"[tw] OR "processing therap*"[tw] OR "affirming care"[tw] OR "empowerment based"[tw] OR "LGBT affirm*"[tw] OR "LGBTQ affirm*"[tw] OR "trans affirm*"[tw] OR "LGBT-affirmative treatment"[tw] OR "behavior therap*"[tw] OR "behaviour therap*"[tw] OR "behavioral therap*"[tw] OR "behavioural therap*"[tw] OR mindfulness[tw] OR "seeking safety"[tw] OR "collaborative care"[tw] OR "stress inoculation training"[tw] OR "present centered therapy"[tw] OR "interpersonal psychotherap*"[tw] OR "acceptance and commitment therapy"[tw] OR "supportive counseling"[tw] OR "supportive therap*"[tw] OR "supportive treatment"[tw] OR "brief intervention*"[tw] OR "scalable intervention*"[tw] OR "integrated care"[tw] OR "cognitive behavioral treatment"[tw] OR "cognitive behavior treatment"[tw] OR "cognitive behavioural treatment"[tw] OR "cognitive behaviour treatment" [tw] OR "prolonged exposure treatment" [tw] OR "dialectical behavioral treatment"[tw] OR "dialectical behaviour treatment"[tw] OR "acceptance and commitment therapy"[tw] OR "acceptance and commitment treatment"[tw] OR "behavior treatment"[tw] OR "behavioral treatment"[tw] OR "behavioural treatment"[tw] OR "behaviour treatment"[tw] OR "brief eclectic treatment"[tw] OR "flooding"[tw] OR "implosive"[tw] OR "present-centered therapy"[tw] OR "cognitive restructuring"[tw] OR "trauma-related guilt reduction"[tw] OR "adapted disclosure"[tw] OR "empowering queer identities in psychotherapy"[tw] OR "effective skills to empower effective men"[tw] OR "virtual reality"[tw] OR "DBT-PE"[tw] OR "interpersonal psychotherapy"[tw] OR "accelerated resolution therapy"[tw] OR "accelerated resolution treatment"[tw] OR "imagery rehearsal therapy"[tw] OR "imagery rehearsal treatment"[tw] OR "trauma management therapy"[tw] OR "trauma management treatment"[tw] OR "processing treatment"[tw] OR "trauma therapy"[tw])) AND (("Trauma and Stressor Related Disorders"[Mesh] OR "Stress Disorders, Post-Traumatic"[Mesh] OR PTSD[tw] OR posttrauma*[tw] OR trauma*[tw] OR "Criterion A stressor*"[tw] OR PTS[tw] OR avoidance[tw] OR "traumatic intrusion*"[tw] OR "intrusive thought*"[tw] OR "intrusive cognition*"[tw] OR "intrusive memor*"[tw] OR hyperarousal[tw] OR hypervigilenc*[tw] OR "post-trauma*"[tw] OR "traumatic injury"[tw] OR "acute stress disorder*"[tw] OR "moral injur*"[tw] OR reexperiencing[tw] OR "re-experiencing"[tw] OR "negative alterations in cognit*" [tw] OR "negative alteration in mood"[tw] OR "fear structure*"[tw])) AND (("Stakeholder Participation"[Mesh] OR "Treatment Outcome"[Mesh] OR "Program Evaluation"[Mesh] OR "Quality Improvement"[Mesh] OR "Implementation Science"[Mesh] OR "treatment outcome*" [mesh] OR effective*[tw] OR efficac*[tw] OR feasibility[tw] OR acceptability[tw] OR implementation[tw] OR sustainability[tw] OR adoption[tw] OR appropriateness[tw] OR fidelity[tw] OR "implementation cost*"[tw] OR "patient experience*"[tw] OR "symptom reduction*"[tw] OR applicability[tw] OR "quality improvement"[tw] OR "program evaluation"[tw] OR "provider perspective*"[tw] OR "provider interview*"[tw] OR "stakeholder perspective*"[tw] OR "stakeholder interview*"[tw] OR "stakeholder feedback"[tw] OR "provider feedback"[tw] OR "end-user input"[tw] OR "symptom change*"[tw] OR "PCL-5"[tw] OR "PTSD checklist"[tw] OR "randomized control trial"[tw] OR "randomized controlled trial*"[tw] OR RCT[tw] OR "pilot trial"[tw] OR "pilot study"[tw] OR review[tw] OR "treatment recommendation*"[tw] OR "clinical recommendation*"[tw] OR "treatment consideration*"[tw] OR "treatment recommendation*"[tw] OR "case series"[tw] OR "case stud*"[tw] OR "clinical trial*"[tw] OR "participatory research"[tw] OR "comparative effectiveness"[tw] OR "comparative efficacy"[tw] OR "case conceptualization*"[tw] OR "case formulation*"[tw] OR qualitative[tw] OR "participatory design"[tw] OR "human-centered design"[tw] OR "human centered design"[tw] OR "community engage*"[tw] OR "focus group*"[tw] OR "focus-group*"[tw] OR "clinical commentar*"[tw] OR editorial*[tw] OR "clinical consideration*"[tw])) AND english [lang] AND 2000:3000 [dp])

**Web of Science Search String**

WOS PASTEABLE SEARCH – Choose 6 "editions" (see screenshot) Add Date/Language filters post-search. Paste search into *Advanced Search/Query Preview.*

(TS=(bisexual* OR F2M OR ("female-to-male" AND gender) OR gay OR gays OR "gender dysphori*" OR "gender identit*" OR "gender queer" OR genderqueer OR GLBQ* OR GLBT* OR homosexual* OR intersex OR lesbian* OR lesbigay OR LGB OR LGBT* OR M2F OR MtoF OR FtoM OR ("male-to-female" AND gender) OR queer* OR "sexual and gender minorit*" OR "sexual identit*" OR "sexual minorit*" OR "sexual orientation*" OR "sexual preference*" OR "trans fem*" OR "trans male" OR "transmasc*" OR "trans man" OR "trans men" OR "trans people" OR "trans wom*" OR transgender* OR transexual* OR "bi-umbrella" OR "trans umbrella" OR "trans and gender divers*" OR "gender divers*" OR TGNC OR "non-binary" OR nonbinary OR "gender nonconforming" OR "gender minorit*" OR BGLT* OR pansexual* OR asexual* OR omnisexual* OR "gender fluid*" OR genderfluid* OR "men loving men" OR "women loving women" OR "two spirit" OR aromantic* OR "grey sexual*" OR demisexual* OR demiromantic* OR "sexual minorit*" OR "same sex" OR "same gender" OR agender* OR sapphic OR "gender euphori*" OR panromantic* OR homoromantic* OR biromantic* OR bigender* OR "asexual umbrella" OR "gender divergen*" OR "gender atypical*" OR "gender expression*" OR butch OR femme OR "trans masc*" OR transfem* OR twink* OR "stud community" OR blaQ OR blaQueer OR "gender expansive*" OR "gender variant" OR "masculine of center" OR "feminine of center" OR "multi sexual*" "multisexual*" OR neutrois OR omnigender* OR "third gender*" OR polygender* "poly gender*" OR pangender* OR "pan gender*" OR polysexual* OR "poly sexual*" OR "romantic orientation*" OR sogie OR "sexual orientation" OR "gender identity" OR "gender expression" OR enby)) AND ((TS=(PTSD OR posttrauma* OR trauma* OR "Criterion A stressor*" OR PTS OR avoidance OR "traumatic intrusion*" OR "intrusive thought*" OR "intrusive cognition*" OR "intrusive memor*" OR hyperarousal OR hypervigilenc* OR "post-trauma*" OR "traumatic injury" OR "acute stress disorder*" OR "moral injur*" OR reexperiencing OR "re-experiencing" OR "negative alterations in cognit*" OR "negative alteration in mood" OR "fear structure*")) OR (TS=("minority stress" OR microaggress* OR concealment OR "concealable identit*" OR discrimination OR homophobia OR biphobia OR transphobia OR harassment OR victimization OR heteronorm* OR homonorm* OR "hate crim*" OR prejudice* OR systemic* OR barriers OR "expectations of rejection" OR "expectation of rejection" OR tokenism OR tokeniz* OR minoritiz* OR marginaliz* OR bias OR burden OR "micro-aggress*" OR macroaggress* OR "macro-aggress*" OR rejection OR lesbophobia OR heterosexis* OR cissexis* OR stigma* OR "traumatic invalidation" OR "family rejection" OR oppression OR oppressive OR outness OR stereotyp*))) AND (TS=(Intervention OR "cognitive behavioral therap*" OR "cognitive behavior therap*" OR "cognitive behavioural therap*" OR "cognitive behaviour therap*" OR "TF-CBT" OR "trauma-focused CBT" OR "trauma focused" OR "prolonged exposure therapy" OR "written exposure" OR "cognitive processing therapy" OR CPT OR "exposure therap*" OR psychotherap* OR "talk therap*" OR "cognitive restructur*" OR "cognitive reappraisal" OR "exposure based" OR "trauma informed" OR "evidence based" OR STAIR OR "STAIR narrative therapy" OR "narrative exposure" OR "narrative therapy" OR "peer support*" OR "therapist led" OR "therapist support*" OR "skills coach" OR "dialectical behavioral therapy" OR "dialectical behavior therapy" OR "dialectical behavioural therapy" OR "dialectical behaviour therapy" OR "cognitive therap*" OR "brief eclectic psychotherapy" OR "empirically supported" OR "trauma treatment" OR "relaxation training" OR "skills training and affective interpersonal regulation" OR EMDR OR "eye movement desensitization reprocessing" OR "eye movement desensitization and reprocessing" OR "processing therap*" OR "affirming care" OR "empowerment based" OR "LGBT affirm*" OR "LGBTQ affirm*" OR "trans affirm*" OR "LGBT-affirmative treatment" OR "behavior therap*" OR "behaviour therap*" OR "behavioral therap*" OR "behavioural therap*" OR mindfulness OR "seeking safety" OR "collaborative care" OR "stress inoculation training" OR "present centered therapy" OR "interpersonal psychotherap*" OR "acceptance and commitment therapy" OR "supportive counseling" OR "supportive therap*" OR "supportive treatment" OR "brief intervention*" OR "scalable intervention*" OR "integrated care" OR "cognitive behavioral treatment" OR "cognitive behavior treatment" OR "cognitive behavioural treatment" OR "cognitive behaviour treatment" OR "prolonged exposure treatment" OR "dialectical behavioral treatment" OR "dialectical behaviour treatment" OR "acceptance and commitment therapy" OR "acceptance and commitment treatment" OR "behavior treatment" OR "behavioral treatment" OR "behavioural treatment" OR "behaviour treatment" OR "brief eclectic treatment" OR "flooding" OR "implosive" OR "present-centered therapy" OR "cognitive restructuring" OR "trauma-related guilt reduction" OR "adapted disclosure" OR "empowering queer identities in psychotherapy" OR "effective skills to empower effective men" OR "virtual reality" OR "DBT-PE" OR "interpersonal psychotherapy" OR "accelerated resolution therapy" OR "accelerated resolution treatment" OR "imagery rehearsal therapy" OR "imagery rehearsal treatment" OR "trauma management therapy" OR "trauma management treatment" OR "processing treatment" OR "trauma therapy")) AND (TS=("treatment outcome*" OR "program evaluation" OR effective* OR efficac* OR feasibility OR acceptability OR implementation OR sustainability OR adoption OR appropriateness OR fidelity OR "implementation cost*" OR "patient experience*" OR "symptom reduction*" OR applicability OR "quality improvement" OR "program evaluation" OR "provider perspective*" OR "provider interview*" OR "stakeholder perspective*" OR "stakeholder interview*" OR "stakeholder feedback" OR "provider feedback" OR "end-user input" OR "symptom change*" OR "PCL-5" OR "PTSD checklist" OR "randomized control trial" OR "randomized controlled trial*" OR RCT OR "pilot trial" OR "pilot study" OR review OR "treatment recommendation*" OR "clinical recommendation*" OR "treatment consideration*" OR "treatment recommendation*" OR "case series" OR "case stud*" OR "clinical trial*" OR "participatory research" OR "comparative effectiveness" OR "comparative efficacy" OR "case conceptualization*" OR "case formulation*" OR qualitative OR "participatory design" OR "human-centered design" OR "human centered design" OR "community engage*" OR "focus group*" OR "focus-group*" OR "clinical commentar*" OR editorial* OR "clinical consideration*"))


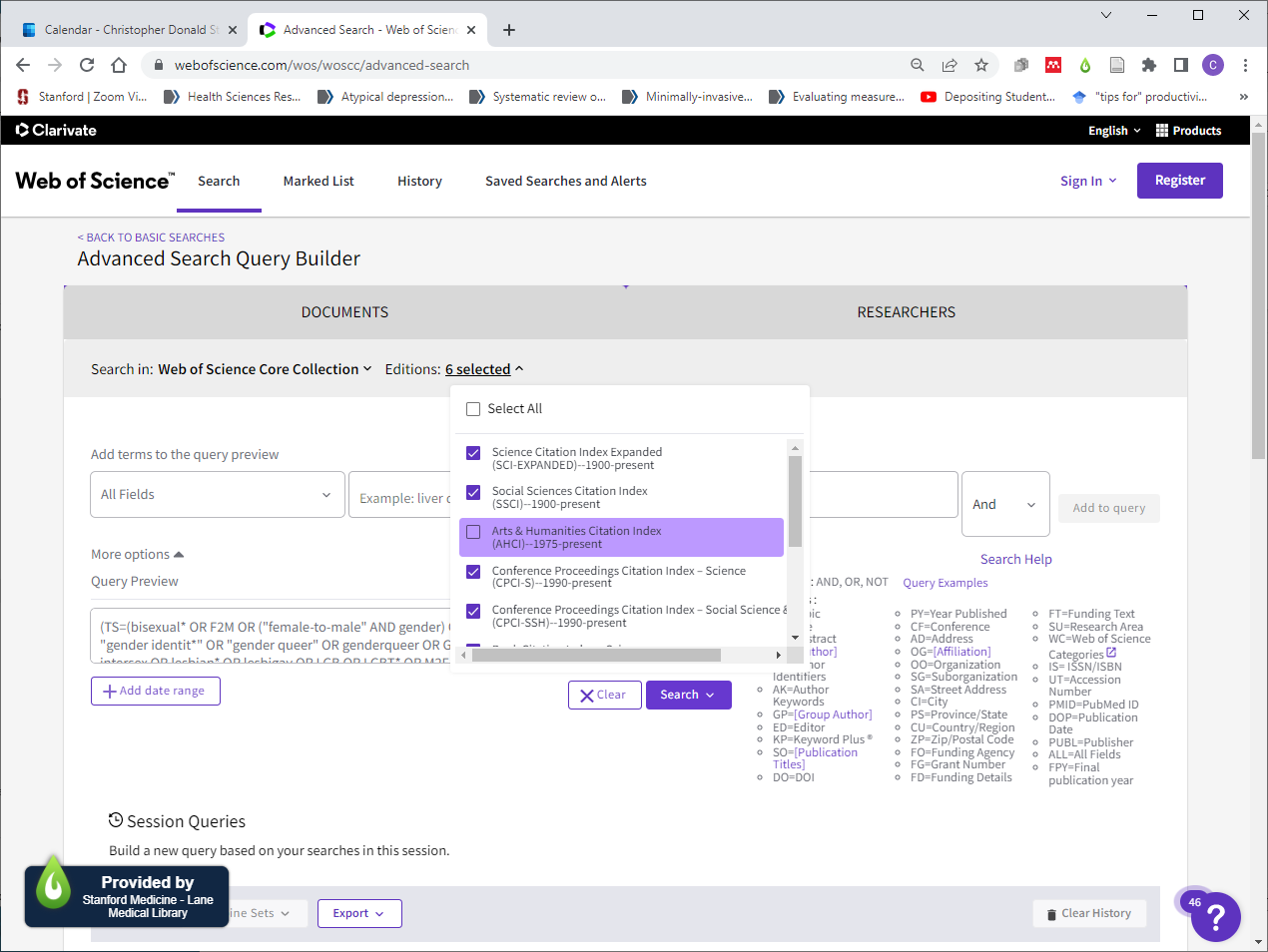


__

1. (TS=(bisexual* OR F2M OR ("female-to-male" AND gender) OR gay OR gays OR "gender dysphori*" OR "gender identit*" OR "gender queer" OR genderqueer OR GLBQ* OR GLBT* OR homosexual* OR intersex OR lesbian* OR lesbigay OR LGB OR LGBT* OR M2F OR MtoF OR FtoM OR ("male-to-female" AND gender) OR queer* OR "sexual and gender minorit*" OR "sexual identit*" OR "sexual minorit*" OR "sexual orientation*" OR "sexual preference*" OR "trans fem*" OR "trans male" OR "transmasc*" OR "trans man" OR "trans men" OR "trans people" OR "trans wom*" OR transgender* OR transexual* OR "bi-umbrella" OR "trans umbrella" OR "trans and gender divers*" OR "gender divers*" OR TGNC OR "non-binary" OR nonbinary OR "gender nonconforming" OR "gender minorit*" OR BGLT* OR pansexual* OR asexual* OR omnisexual* OR "gender fluid*" OR genderfluid* OR "men loving men" OR "women loving women" OR "two spirit" OR aromantic* OR "grey sexual*" OR demisexual* OR demiromantic* OR "sexual minorit*" OR "same sex" OR "same gender" OR agender* OR sapphic OR "gender euphori*" OR panromantic* OR homoromantic* OR biromantic* OR bigender* OR "asexual umbrella" OR "gender divergen*" OR "gender atypical*" OR "gender expression*" OR butch OR femme OR "trans masc*" OR transfem* OR twink* OR "stud community" OR blaQ OR blaQueer OR "gender expansive*" OR "gender variant" OR "masculine of center" OR "feminine of center" OR "multi sexual*" "multisexual*" OR neutrois OR omnigender* OR "third gender*" OR polygender* "poly gender*" OR pangender* OR "pan gender*" OR polysexual* OR "poly sexual*" OR "romantic orientation*" OR sogie OR "sexual orientation" OR "gender identity" OR "gender expression" OR enby)) AND ((TS=(PTSD OR posttrauma* OR trauma* OR "Criterion A stressor*" OR PTS OR avoidance OR "traumatic intrusion*" OR "intrusive thought*" OR "intrusive cognition*" OR "intrusive memor*" OR hyperarousal OR hypervigilenc* OR "post-trauma*" OR "traumatic injury" OR "acute stress disorder*" OR "moral injur*" OR reexperiencing OR "re-experiencing" OR "negative alterations in cognit*" OR "negative alteration in mood" OR "fear structure*")) OR (TS=("minority stress" OR microaggress* OR concealment OR "concealable identit*" OR discrimination OR homophobia OR biphobia OR transphobia OR harassment OR victimization OR heteronorm* OR homonorm* OR "hate crim*" OR prejudice* OR systemic* OR barriers OR "expectations of rejection" OR "expectation of rejection" OR tokenism OR tokeniz* OR minoritiz* OR marginaliz* OR bias OR burden OR "micro-aggress*" OR macroaggress* OR "macro-aggress*" OR rejection OR lesbophobia OR heterosexis* OR cissexis* OR stigma* OR "traumatic invalidation" OR "family rejection" OR oppression OR oppressive OR outness OR stereotyp*))) AND (TS=(Intervention OR "cognitive behavioral therap*" OR "cognitive behavior therap*" OR "cognitive behavioural therap*" OR "cognitive behaviour therap*" OR "TF-CBT" OR "trauma-focused CBT" OR "trauma focused" OR "prolonged exposure therapy" OR "written exposure" OR "cognitive processing therapy" OR CPT OR "exposure therap*" OR psychotherap* OR "talk therap*" OR "cognitive restructur*" OR "cognitive reappraisal" OR "exposure based" OR "trauma informed" OR "evidence based" OR STAIR OR "STAIR narrative therapy" OR "narrative exposure" OR "narrative therapy" OR "peer support*" OR "therapist led" OR "therapist support*" OR "skills coach" OR "dialectical behavioral therapy" OR "dialectical behavior therapy" OR "dialectical behavioural therapy" OR "dialectical behaviour therapy" OR "cognitive therap*" OR "brief eclectic psychotherapy" OR "empirically supported" OR "trauma treatment" OR "relaxation training" OR "skills training and affective interpersonal regulation" OR EMDR OR "eye movement desensitization reprocessing" OR "eye movement desensitization and reprocessing" OR "processing therap*" OR "affirming care" OR "empowerment based" OR "LGBT affirm*" OR "LGBTQ affirm*" OR "trans affirm*" OR "LGBT-affirmative treatment" OR "behavior therap*" OR "behaviour therap*" OR "behavioral therap*" OR "behavioural therap*" OR mindfulness OR "seeking safety" OR "collaborative care" OR "stress inoculation training" OR "present centered therapy" OR "interpersonal psychotherap*" OR "acceptance and commitment therapy" OR "supportive counseling" OR "supportive therap*" OR "supportive treatment" OR "brief intervention*" OR "scalable intervention*" OR "integrated care" OR "cognitive behavioral treatment" OR "cognitive behavior treatment" OR "cognitive behavioural treatment" OR "cognitive behaviour treatment" OR "prolonged exposure treatment" OR "dialectical behavioral treatment" OR "dialectical behaviour treatment" OR "acceptance and commitment therapy" OR "acceptance and commitment treatment" OR "behavior treatment" OR "behavioral treatment" OR "behavioural treatment" OR "behaviour treatment" OR "brief eclectic treatment" OR "flooding" OR "implosive" OR "present-centered therapy" OR "cognitive restructuring" OR "trauma-related guilt reduction" OR "adapted disclosure" OR "empowering queer identities in psychotherapy" OR "effective skills to empower effective men" OR "virtual reality" OR "DBT-PE" OR "interpersonal psychotherapy" OR "accelerated resolution therapy" OR "accelerated resolution treatment" OR "imagery rehearsal therapy" OR "imagery rehearsal treatment" OR "trauma management therapy" OR "trauma management treatment" OR "processing treatment" OR "trauma therapy")) AND (TS=("treatment outcome*" OR "program evaluation" OR effective* OR efficac* OR feasibility OR acceptability OR implementation OR sustainability OR adoption OR appropriateness OR fidelity OR "implementation cost*" OR "patient experience*" OR "symptom reduction*" OR applicability OR "quality improvement" OR "program evaluation" OR "provider perspective*" OR "provider interview*" OR "stakeholder perspective*" OR "stakeholder interview*" OR "stakeholder feedback" OR "provider feedback" OR "end-user input" OR "symptom change*" OR "PCL-5" OR "PTSD checklist" OR "randomized control trial" OR "randomized controlled trial*" OR RCT OR "pilot trial" OR "pilot study" OR review OR "treatment recommendation*" OR "clinical recommendation*" OR "treatment consideration*" OR "treatment recommendation*" OR "case series" OR "case stud*" OR "clinical trial*" OR "participatory research" OR "comparative effectiveness" OR "comparative efficacy" OR "case conceptualization*" OR "case formulation*" OR qualitative OR "participatory design" OR "human-centered design" OR "human centered design" OR "community engage*" OR "focus group*" OR "focus-group*" OR "clinical commentar*" OR editorial* OR "clinical consideration*"))

**SUPPLEMENT B: ABSTRACT/TITLE SCREENING AND FULL-TEXT REVIEW INSTRUCTIONS**

**Updated: 2/24/23**

**Sexual and Gender Minority Stress and PTSD Scoping Review**

**Title/Abstract Screening Instructions**

***Accessing assigned references***

1. Open tracker > navigate to your name > find the **covnum** for the reference in **column A**
2. Open Covidence <https://app.covidence.org/reviews/229637> and click "search studies" in the upper right corner
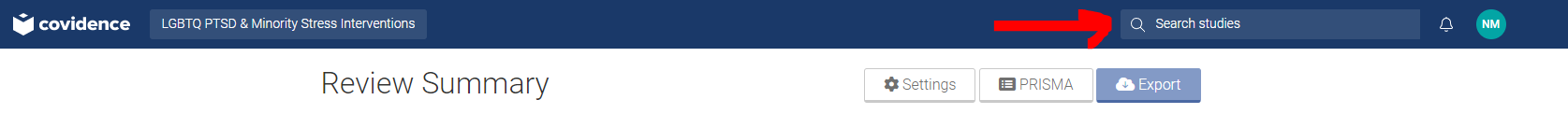

3. Type in the covnum of your assigned article and it should open the abstract. For example, when I typed #1 in the "search studies" box, this came up:


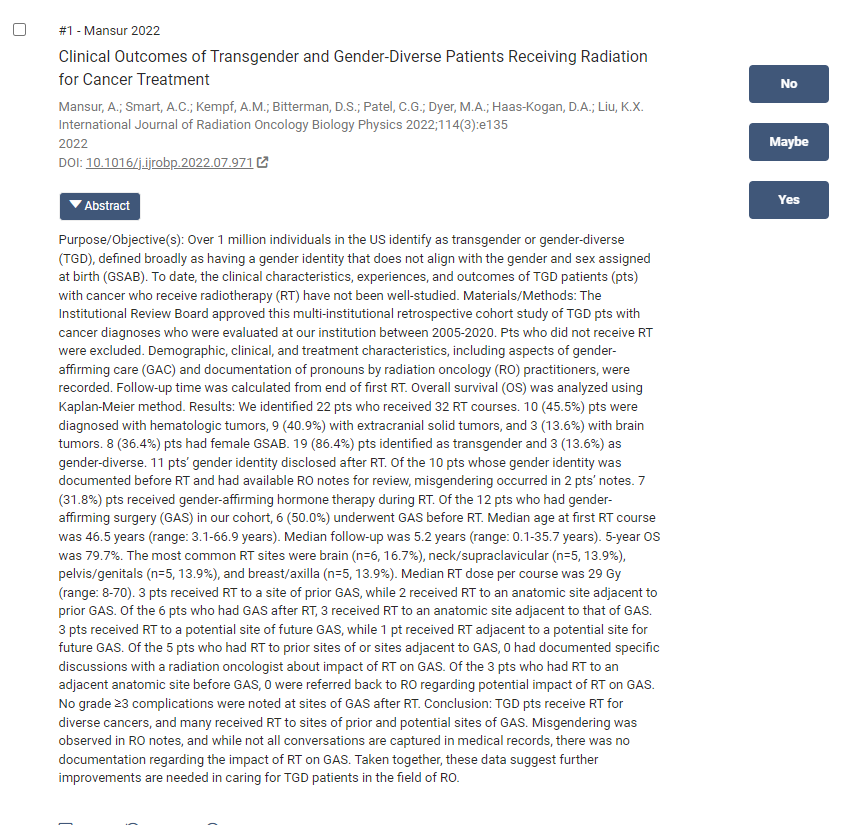


NOTE: You need to add the number sign (#) before the number in order for it to properly search the study you are looking for.

***Reviewing assigned references***

***NOTE: In each abstract, click "show criteria" in the bar above the abstract you are reviewing to see the same table of criteria as below.**
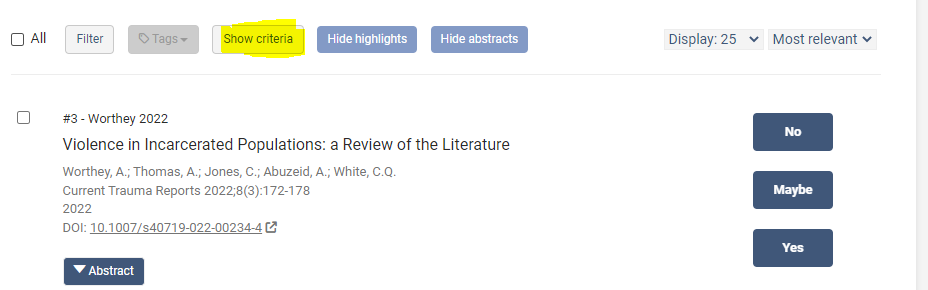


| **Inclusion Criteria** | **Exclusion Criteria** |
| --- | --- |
| Year of publication: 2000-present |  |
| Language: English |  |
| Population: Sexual and gender minority adults (if mixed sample with some participants age ≥ 18, then include); human |  |
| Intervention: Any psychological, psychotherapeutic, or behavioral intervention targeting trauma, PTSD, or minority stress | Intervention: "conversion" therapy; medication only; surgical only; hormonal only |
| Publication type: peer-reviewed reviews, peer-reviewed research articles, unpublished dissertations/theses, peer-reviewed clinical recommendations, unpublished conference papers | Publication type: letters to the editor, non-peer reviewed journals, blogs, magazines, book chapters, book reviews, retracted publications, treatment manuals, introductions to special issues |
| Design: case series, case studies, quality improvement, stakeholder feedback, clinical trials, theoretical papers introducing a new treatment |  |
| Outcomes: PTSD symptoms; minority stress; concealment; internalized stigma; experiences of discrimination; expectations for discrimination; resilience factors; client/patient satisfaction; implementation science outcomes (e.g., feasibility, acceptability, cost)  *Note: minority stress includes the following four components. Inclusion of any of these components is sufficient to meet the minority stress outcome criteria.  1. internalized stigma, e.g., internalized forms of homophobia, biphobia, transphobia, heterosexism, cissexism, etc.  2. experiences of discrimination, e.g., from providers, family, friends, coworkers, etc.  3. expectations of discrimination, e.g., from providers, family, friends, coworkers, etc.  4. concealment, e.g., outtness, management of concealable identity, "passing" | Outcomes: public health outcomes only (e.g., safe sex) |

1. The options listed to the right of each abstract are: No, Maybe, Yes. Refer to the inclusion/exclusion criteria above when making these decisions. As a reminder, you can also click "show criteria" to see the inclusion/exclusion criteria.
   1. **No** = does not meet inclusion criteria; excluded
   2. **Maybe** = might fit with our inclusion criteria but hard to say at the title/abstract stage, should go to full-text review to decide
   3. **Yes** = meets screening criteria based on title/abstract; included for full-text review
2. Covidence has a feature that allow you to have certain inclusion/exclusion terms show up as "highlights". See below for our list our highlight terms.


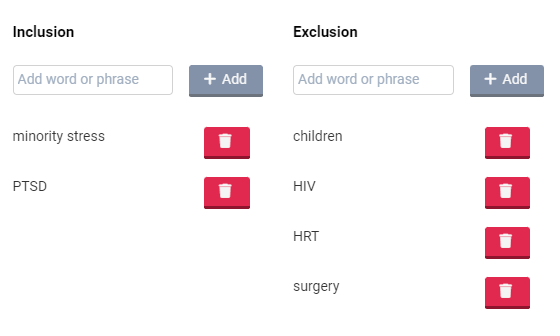


2a. In the abstract you are reviewing, click on "show highlights". This will highlight the terms from the list above that are in the abstract you are reviewing. Keep in mind that this is a helpful guide but it DOES NOT necessarily help on its own decide if it meets inclusion/exclusion criteria. Be sure to review the whole abstract before deciding your rating.


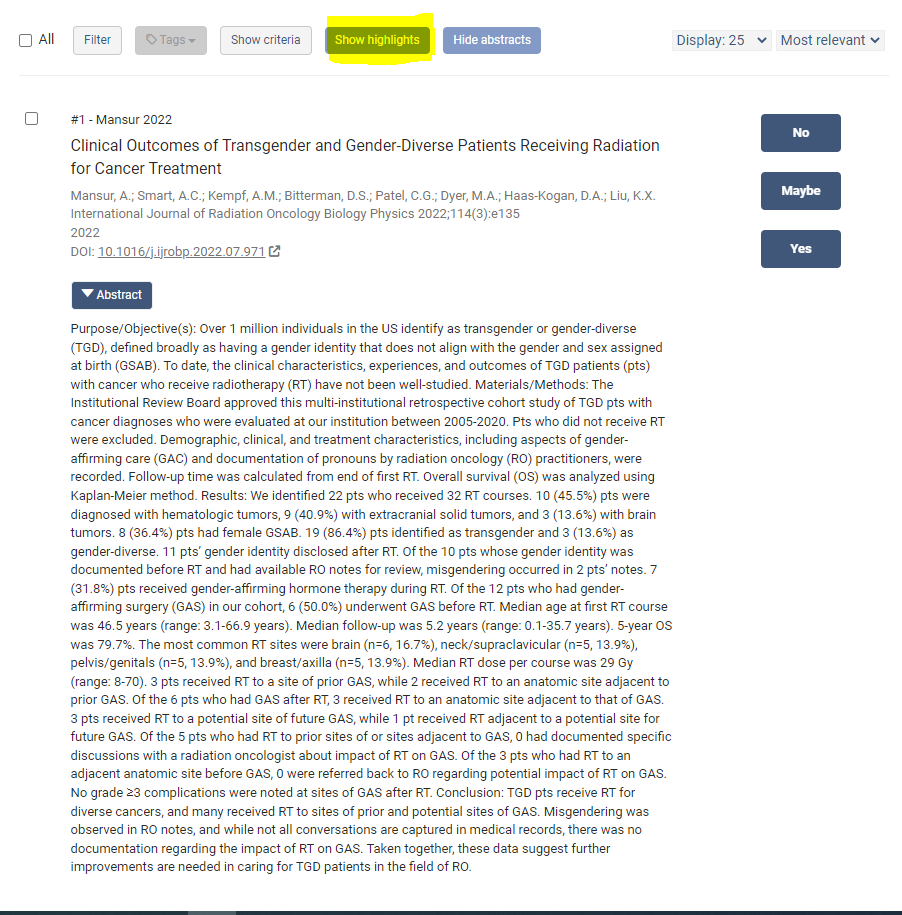


1. We have also added a "tag" in covidence titled "digital intervention." When you come across an abstract that pertains to digital interventions, mHealth, wearables, etc… please make sure to use the digital intervention tag (regardless of inclusion/exclusion criteria)
   1. Above each abstract, you will see this bar of options. Check the box that says "all"


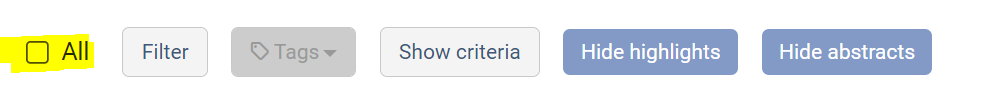


- 1. Then, click the "tags" tab so the dropdown below appears and then select "digital intervention"


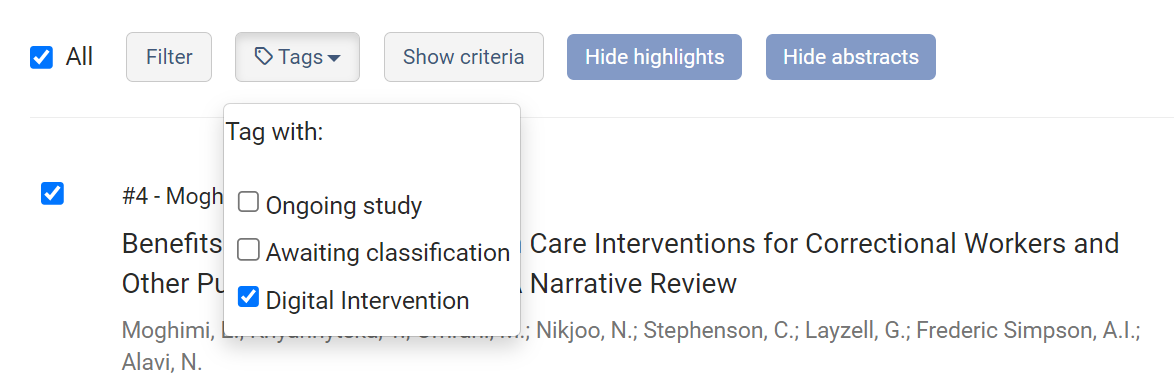


1. Once you have done your rating, mark the date you completed it in the appropriate column in the tracker. Do not add your ratings to the tracker.

**Sexual and Gender Minority Stress and PTSD Scoping Review**

**Full-Text Review Instructions**

1. Open full-text review tracker in GoogleDocs > navigate to your name > find the **covnum** for the reference in **column A**
2. Open Covidence <https://app.covidence.org/reviews/229637>
3. Login with your associated email and password, and click the associated project (LGBTQ PTSD & Minority Stress Interventions) if this option populates.
4. From the dashboard scroll down and extend the arrow next to Full text review to see the status of the review, as shown below:


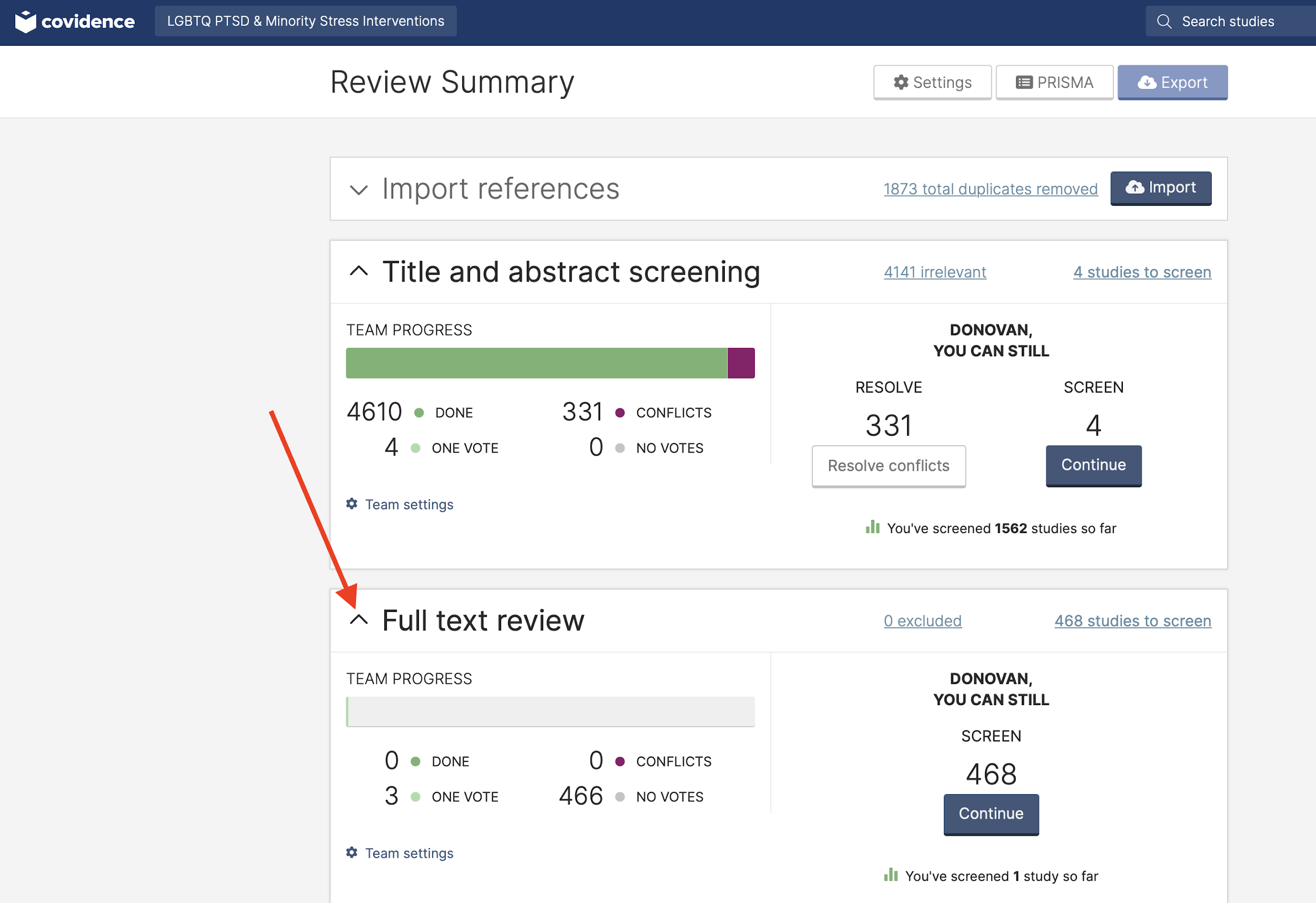


1. Click continue and type in # and the covnum from column A of your assigned articles (e.g, #1). **The article will not populate without the # symbol.** This will populate the associated article. t should open the abstract. For example, if you were to type #947 in the "search studies" box, the article will populate:


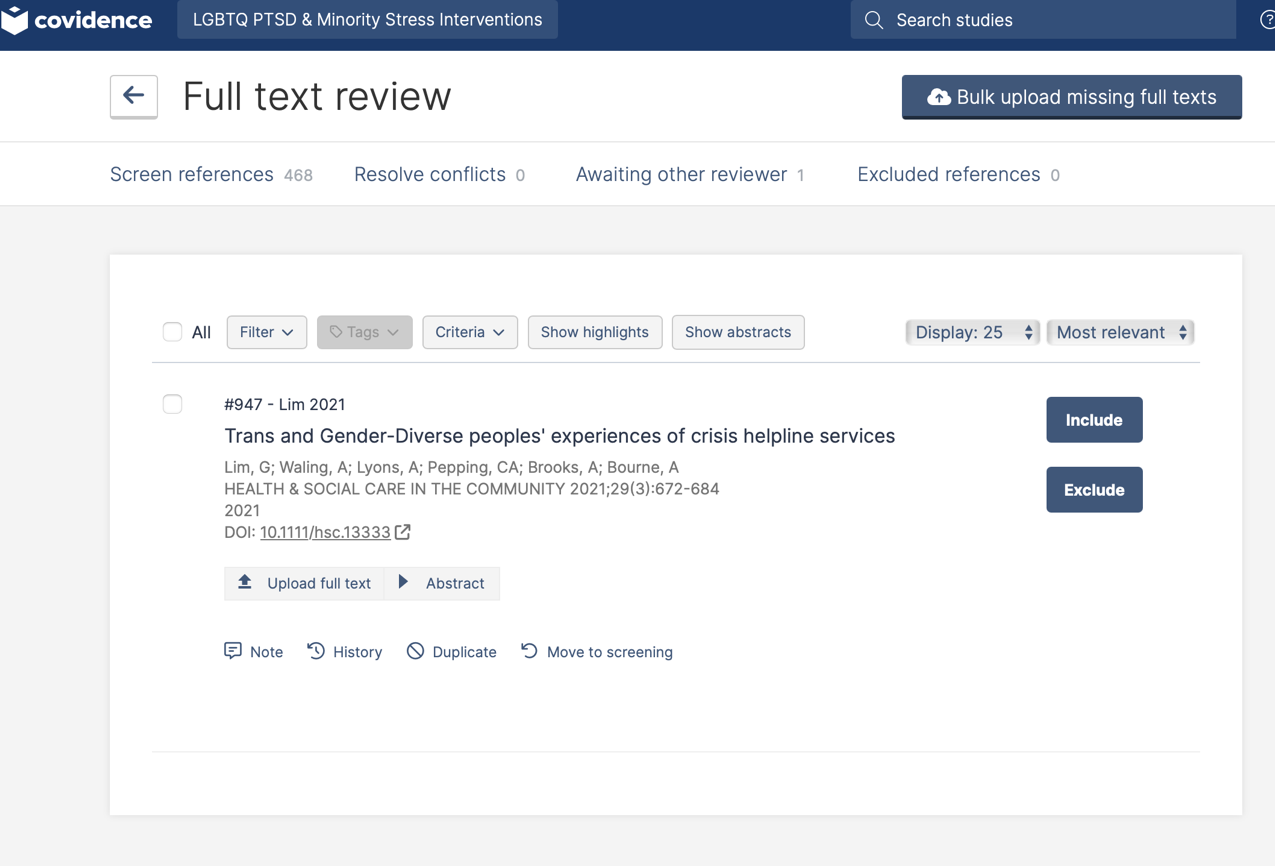


1. From this point, you have the option to review the abstract and read the full text. Please read both. To do so, click either **Abstract** or the **Hyperlink beside the DOI:**
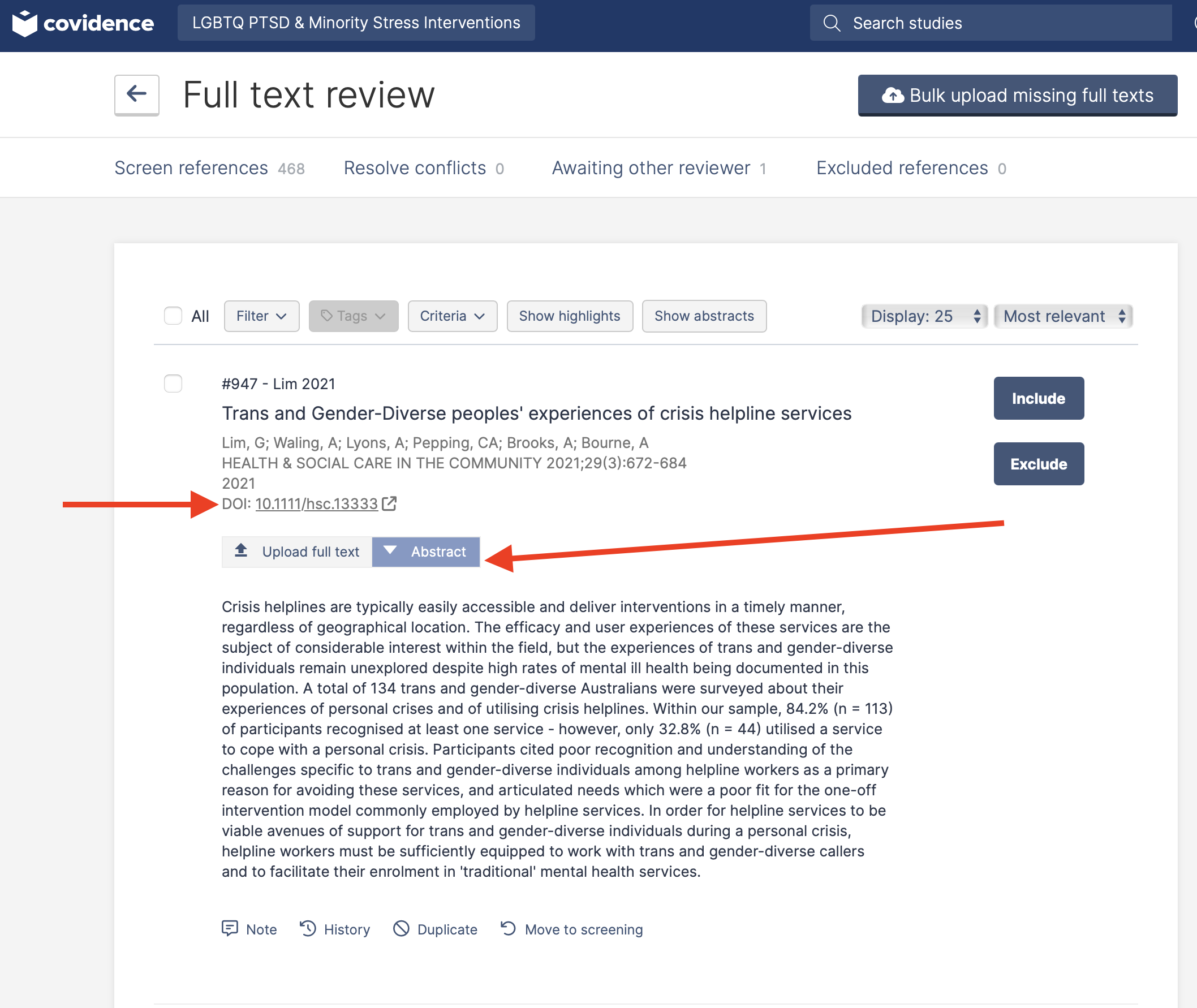

2. The hyperlink for full text will create an additional tab for the article and take you to a page such as this:
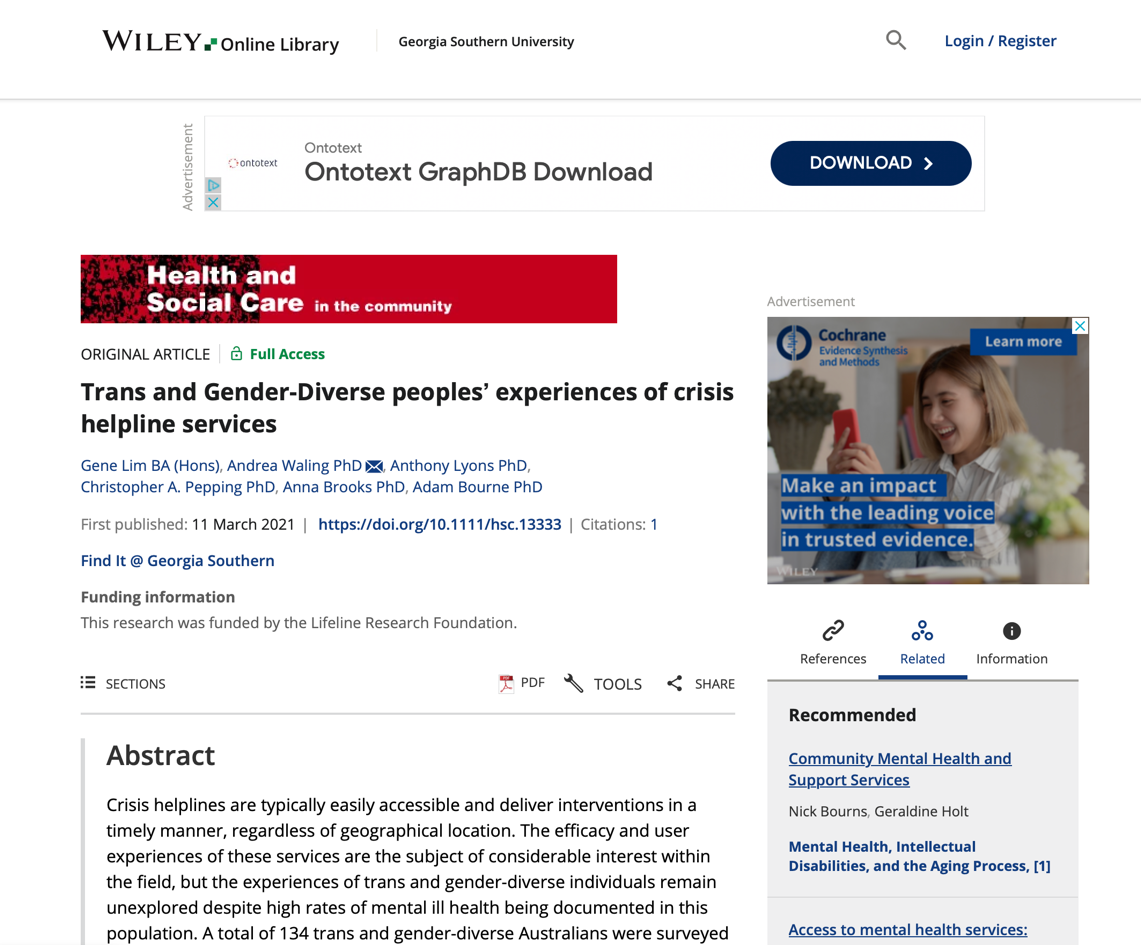

3. In some cases, the full article will not be available to review. For these times, please search the article on the following websites:
   1. [Google Scholar](https://scholar.google.com/) is a free resource that will often have a full-text PDF accessible, such as in the image below:
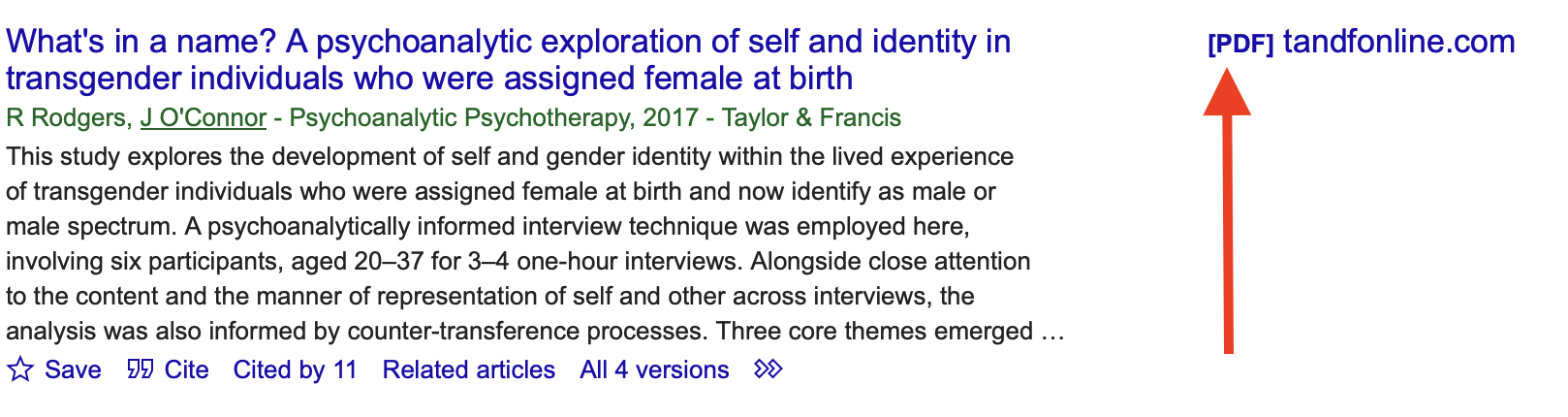

   2. Websites such as PsycArticles, PsycInfo, or other locations that house full-text articles are accessible through many university intranets and library requests. You may need to wait for an article to be delivered through your academic affiliate library. Please place requests and mark that you are waiting for the full-text in the spreasheet (“W” = leave a W in the date column to indicate that you are waiting for the full-text). Then, move on to your next assigned full-text review, and return to the assigned reference when you have the full-text available.
4. Once you have found the full-text article, download the article and return to the article location on Covidence. Click the **Upload full text prompt,** and either **Choose a file** to upload or **drag and drop** the file. Then click Finish.


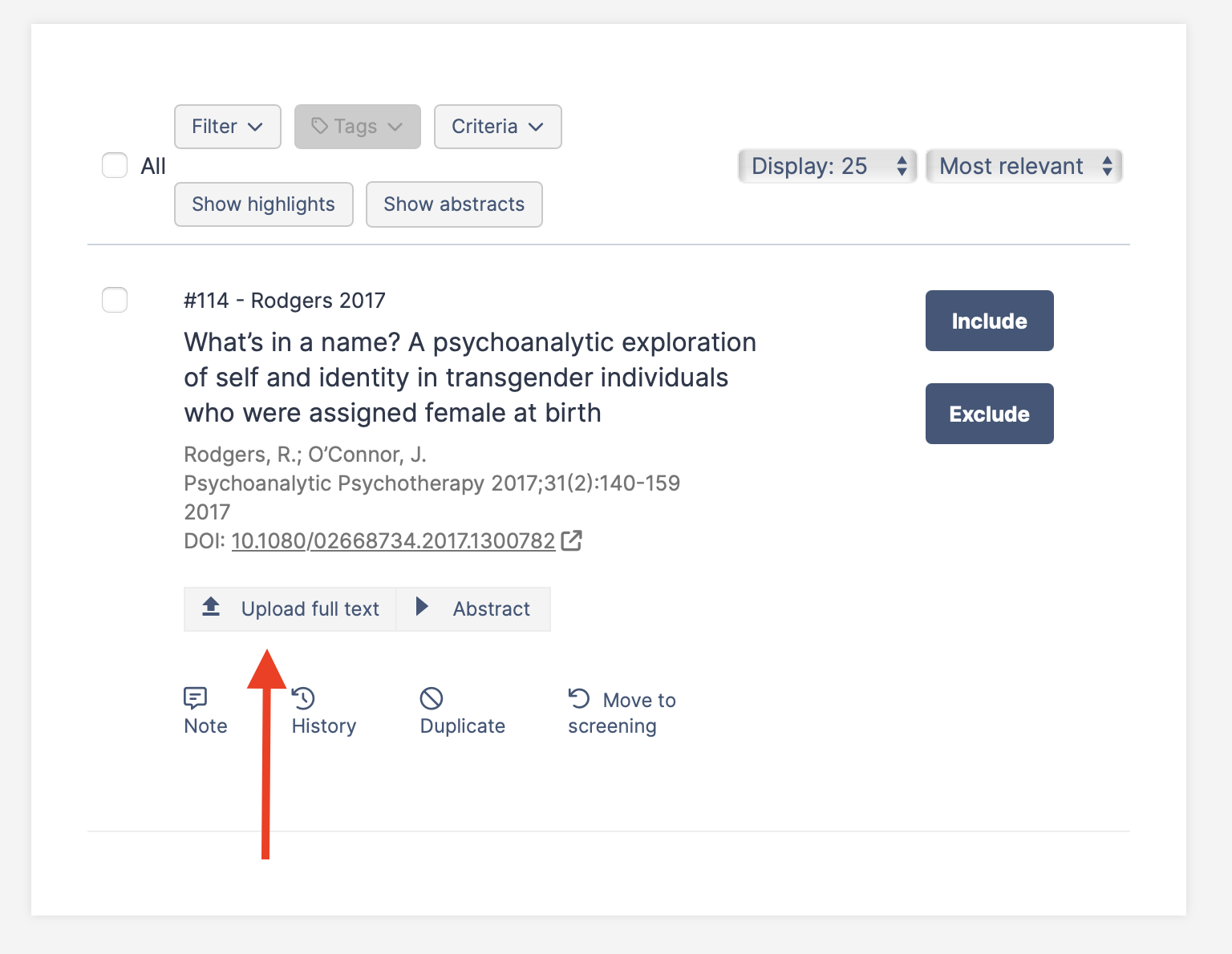


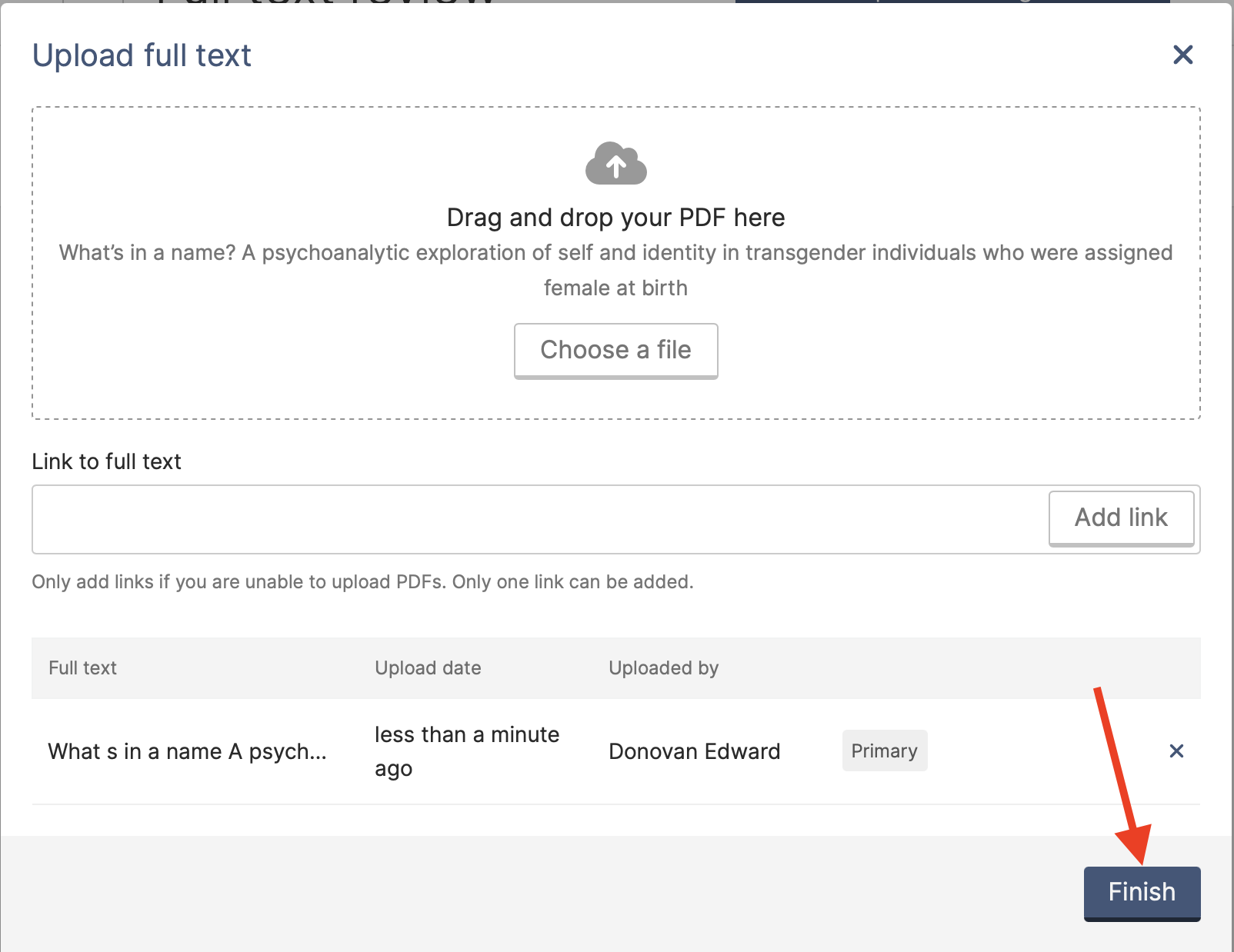


1. The options listed to the right of each abstract are: Include and Exclude. Refer to the inclusion/exclusion criteria below when making these decisions. **For all excluded works, please ensure that you select the relevant reason(s) for exclusion.**
   1. **Include** = meets inclusion criteria; included
   2. **Exclude** = does not meet inclusion criteria or meets exclusion criteria; excluded
   3. **IMPORTANT TIPS:**
      1. **In order to include a work, trauma, PTSD, and/or minority stress must be a main focus.**
      2. **We are interested in intervention-focused works. This can include original intervention research, clinical commentaries, etc.**
      3. **For original research papers, we are interested ONLY in only intervention-focused studies. The research question must be related to developing, testing, or modifying a trauma, PTSD, and/or minority stress intervention for LGBTQ+ adults**
      4. **For treatment outcomes research, at least one of the outcomes must be TRAUMA SYMPTOMS OR PTSD symptoms (or diagnostic status) OR minority stress symptoms.**
      5. **For review papers and meta-analyses, at least one of the studies reviewed should meet our eligibility criteria**

| **Inclusion Criteria** | **Exclusion Criteria** |
| --- | --- |
| Year of publication: 2000-present | Year of publication: 1999 or earlier |
| Language: English |  |
| Population: Sexual and gender minority individuals- this can be broad (e.g., LGBTQIA+ adults) or specific (e.g., Latino gay men, transgender adults, bisexual adults, LGB older adults, Black lesbians); human; adults*  *Note: Only include studies with “youths” if age range includes individuals 18 years or older) | Population: Children*  *Note: Only exclude studies with “youths” if the age range is ≤17 years old. |
| Intervention: Any individual, group, partner/family-supported* psychological, psychotherapeutic, or behavioral intervention targeting trauma, PTSD, or minority stress  *Note: Only include couples and family therapy if the treatment is a partner/family-supported intervention with primary treatment targets of one couple/family member’s trauma, PTSD, or minority stress symptoms. Do not include if primary targets are relationship quality, family functioning, etc. | Intervention: "conversion" therapy; medication only; surgical only; hormonal only; couples therapy*; family therapy*; provider trainings  *Note: Exclude couples and family therapy in general, but do not exclude partner/family-supported interventions with a primary treatment target of one couple/family member’s trauma, PTSD, or minority stress symptoms. Do not include if primary targets are relationship quality, family functioning, etc. |
| Publication type: peer-reviewed reviews; peer-reviewed research articles; unpublished dissertations/theses; peer-reviewed clinical recommendations; unpublished conference papers; clinical commentaries | Publication type: letters to the editor; non-peer reviewed journals; blogs; magazines; book chapters; book reviews; retracted publications; treatment manuals; introductions to special issues, commentaries on previous papers; protocol papers |
| Design: case series, case studies, quality improvement, stakeholder feedback*, clinical trials (including pilot trials, feasibility trials, randomized controlled trials, and other clinical trial designs); theoretical papers introducing a new treatment for trauma, PTSD, and/or minority stress  *Note: Stakeholder feedback should only be included if it is on a specific intervention for trauma, PTSD, or minority stress AND feedback was collected from LGBTQ+ adults (i.e., not just from their providers) | Design: Research question is not intervention-related; stakeholder feedback with provider perspectives only |
| Outcomes: [Only relevant for original research, meta-analyses, and review papers] trauma symptoms; PTSD symptoms (or diagnostic status); minority stress; concealment; internalized stigma*; experiences of discrimination, rejection, or oppression; expectations for discrimination, rejection, or oppression; resilience factors*; client/patient satisfaction/experiences*; implementation science outcomes (e.g., feasibility, acceptability, cost)*; help-seeking or treatment-seeking for trauma, PTSD, or minority stress  *Note: if stigma is the only relevant outcome, it MUST be identity-based LGBTQ+ internalized stigma (e.g., internalized homophobia, internalized cissexism, internalized transphobia); treatments targeting other mental health conditions (e.g., eating disorder, substance use, depression) can be included if the outcomes assessed include one or more of the eligible outcomes listed above; if outcomes do not include trauma symptoms, PTSD symptoms, or minority stress symptoms but do include resilience factors, client/patient satisfaction, or implementation outcomes, the intervention MUST be a specific trauma, PTSD, and/or minority stress intervention | Outcomes: [Only relevant for original research, meta-analyses, and review papers] public health outcomes only (e.g., safe sex, HIV)*; other mental health outcomes only*; only other forms of stigma that are NOT internalized LGBTQ-identity-based stigma (e.g., social stigma, HIV-stigma); minority stress only discussed in context of another condition*; help-seeking or treatment-seeking for a problem other than trauma, PTSD, or minority stress  Note: If stigma is the only relevant outcome, only identity-based LGBTQ+ internalized stigma should be included; If the focus is another disorder but they mention minority stress, do not include if minority stress is only discussed in the context of that disorder (e.g., “it’s important to address minority stress in service of reducing substance abuse”) |

1. Covidence has a feature that allows you to have certain inclusion/exclusion terms show up as "highlights". In the abstract you are reviewing, click on "Show highlights". This will highlight the terms from the list above that are in the abstract you are reviewing. **Keep in mind that this is a helpful guide, but it DOES NOT necessarily help on its own decide if it meets inclusion/exclusion criteria**. Be sure to review the whole text before deciding your rating See below for our list of highlight terms.


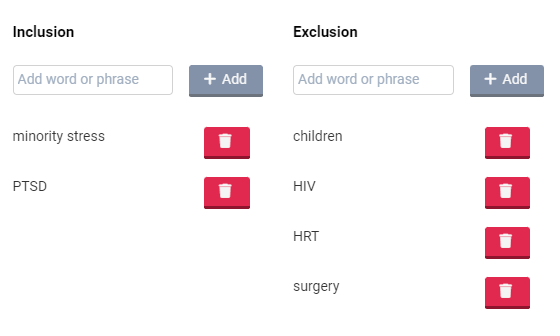


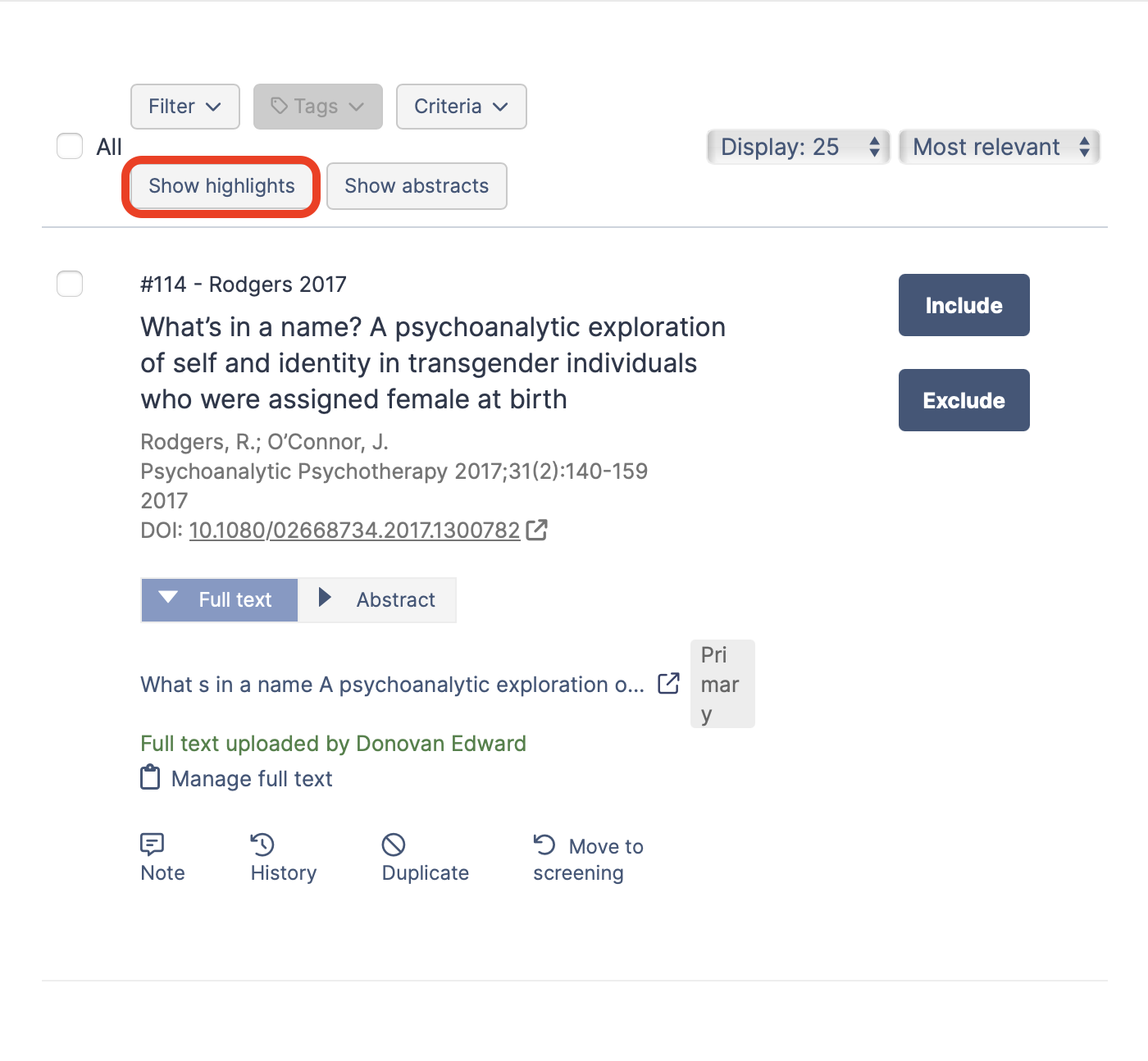


1. Once you have completed your rating, mark the date you completed it in the appropriate column in the tracker. **Do not add your ratings to this tracker.**
